# iSuRe-HadCre is an essential tool for effective conditional genetics

**DOI:** 10.1093/nar/gkae472

**Published:** 2024-06-08

**Authors:** Irene Garcia-Gonzalez, Susana F Rocha, Anahita Hamidi, Lourdes Garcia-Ortega, Alvaro Regano, Maria S Sanchez-Muñoz, Mariya Lytvyn, Aroa Garcia-Cabero, Sergi Roig-Soucase, Hans Schoofs, Marco Castro, Helena Sabata, Michael Potente, Mariona Graupera, Taija Makinen, Rui Benedito

**Affiliations:** Molecular Genetics of Angiogenesis Group, Centro Nacional de Investigaciones Cardiovasculares (CNIC), Madrid, Spain; Molecular Genetics of Angiogenesis Group, Centro Nacional de Investigaciones Cardiovasculares (CNIC), Madrid, Spain; Molecular Genetics of Angiogenesis Group, Centro Nacional de Investigaciones Cardiovasculares (CNIC), Madrid, Spain; Molecular Genetics of Angiogenesis Group, Centro Nacional de Investigaciones Cardiovasculares (CNIC), Madrid, Spain; Molecular Genetics of Angiogenesis Group, Centro Nacional de Investigaciones Cardiovasculares (CNIC), Madrid, Spain; Molecular Genetics of Angiogenesis Group, Centro Nacional de Investigaciones Cardiovasculares (CNIC), Madrid, Spain; Molecular Genetics of Angiogenesis Group, Centro Nacional de Investigaciones Cardiovasculares (CNIC), Madrid, Spain; Molecular Genetics of Angiogenesis Group, Centro Nacional de Investigaciones Cardiovasculares (CNIC), Madrid, Spain; Molecular Genetics of Angiogenesis Group, Centro Nacional de Investigaciones Cardiovasculares (CNIC), Madrid, Spain; Uppsala University, Department of Immunology, Genetics and Pathology, Dag Hammarskjölds väg 20, 751 85 Uppsala, Sweden; Angiogenesis & Metabolism Laboratory, Center of Vascular Biomedicine, Berlin Institute of Health at Charité – Universitätsmedizin Berlin, Berlin, Germany; Max Delbrück Center for Molecular Medicine in the Helmholtz Association, Berlin, Germany; Endothelial Pathobiology and Microenviroment Group, Josep Carreras Leukaemia Research Institute (IJC), 08916 Badalona, Barcelona, Catalonia, Spain; Angiogenesis & Metabolism Laboratory, Center of Vascular Biomedicine, Berlin Institute of Health at Charité – Universitätsmedizin Berlin, Berlin, Germany; Max Delbrück Center for Molecular Medicine in the Helmholtz Association, Berlin, Germany; Endothelial Pathobiology and Microenviroment Group, Josep Carreras Leukaemia Research Institute (IJC), 08916 Badalona, Barcelona, Catalonia, Spain; Centro de Investigación Biomédica en Red de Cáncer (CIBERONC), Instituto de Salud Carlos III, Av. de Monforte de Lemos, 5, 28029 Madrid, Spain; ICREA, Institució Catalana de Recerca i Estudis Avançats, Pg. Lluís Companys 23, Barcelona, Spain; Uppsala University, Department of Immunology, Genetics and Pathology, Dag Hammarskjölds väg 20, 751 85 Uppsala, Sweden; Translational Cancer Medicine Program, Research Programs Unit, Biomedicum Helsinki, University of Helsinki, Haartmaninkatu 8, 00014 Helsinki, Finland; Wihuri Research Institute, Haartmaninkatu 8, 00290 Helsinki, Finland; Molecular Genetics of Angiogenesis Group, Centro Nacional de Investigaciones Cardiovasculares (CNIC), Madrid, Spain

## Abstract

Methods for modifying gene function at high spatiotemporal resolution in mice have revolutionized biomedical research, with Cre-loxP being the most widely used technology. However, the Cre-loxP technology has several drawbacks, including weak activity, leakiness, toxicity, and low reliability of existing Cre-reporters. This is mainly because different genes flanked by loxP sites (floxed) vary widely in their sensitivity to Cre-mediated recombination. Here, we report the generation, validation, and utility of iSuRe-HadCre, a new dual Cre-reporter and deleter mouse line that avoids these drawbacks. iSuRe-HadCre achieves this through a novel inducible dual-recombinase genetic cascade that ensures that cells expressing a fluorescent reporter had only transient Cre activity, that is nonetheless sufficient to effectively delete floxed genes. iSuRe-HadCre worked reliably in all cell types and for the 13 floxed genes tested. This new tool will enable the precise, efficient, and trustworthy analysis of gene function in entire mouse tissues or in single cells.

## Graphical Abstract

**Graphical Abstract:**
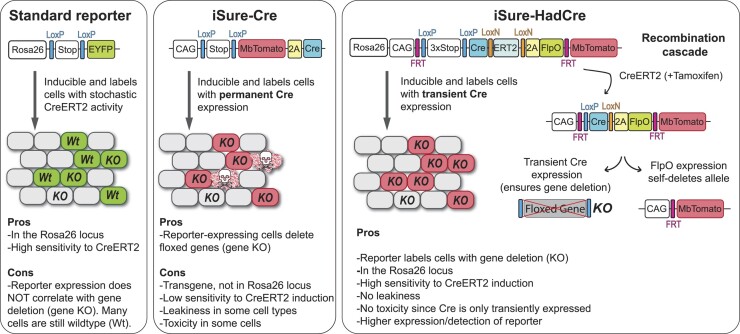

## Introduction

Understanding how genes function requires methods to deplete or enhance their expression, thus allowing assessment of the ensuing phenotypic consequences. The current gold-standard method uses the Cre-Lox technology, which enables precise loss- or gain-of-gene-function (1). However, this technology often produces variable results and requires careful validation of the intended conditional genetic deletions (2,3). This is largely because different floxed genes vary in their sensitivity to Cre-mediated recombination (4,5). Validation of gene deletion involves either whole-organ or cell-type-specific FACS isolations in bulk, qRT-PCR, or western blot analyses with specific probes. However, these approaches lack single cell resolution and the tissues extracted for genetic deletion validation cannot be the same as those used for phenotype or imaging analysis, frequently resulting in ‘genetically blind’ results. The alternative *in situ* validation of protein or mRNA loss after deletion of a floxed gene is often difficult due to lack of good probes or antibodies giving a good signal-noise ratio at single cell resolution, in every cell expressing the gene.

As an alternative, many scientists infer the intended deletion of a floxed gene by using standard Cre-reporters to label cells that recombined the reporter allele. However, recombination of generic Cre-reporters does not correlate with recombination of other floxed genes (6–8), particularly when using tamoxifen-inducible Cre lines, in which recombination activity is weaker and often incomplete, targeting only a fraction of the tissue cells.

We previously generated the *iSure-Cre* allele, that significantly facilitated conditional genetics because it linked the expression of a single and easy to detect fluorescent reporter, to the permanent expression of Cre, which unlike CreERT2, efficiently recombines and deletes floxed genes (7). This guarantees that, irrespective of the level of CreERT2 expression and the intensity of tamoxifen induction, all reporter-expressing cells will have permanent expression of Cre, drastically reducing the occurrence of false positives. False positives occur when cells expressing a given Cre-reporter, do not have recombination of the intended floxed gene, and are a major cause of variability and inaccuracy in biomedical research using mouse genetics. Despite its advantages, the first-generation *iSuRe-Cre* technology also has its caveats, such as relatively low sensitivity to CreERT2 activity, leakiness, and the possibility of toxicity in cells permanently expressing Cre (7).

Here, we present the characterization of several new mouse lines that were designed to overcome these limitations. Only one of these showed a significant improvement compared with the original *iSuRe-Cre* line. This new line, which we call *iSuRe-HadCre*, is several fold brighter, more sensitive to induction by CreERT2/tamoxifen, has no leakiness, and ensures that Cre is only transiently expressed, thus preventing Cre-related toxicity. *iSuRe-HadCre* is based on a new tight and inducible recombination cascade that first converts inducible CreERT2 activity into constitutive Cre expression, followed by an inbuilt FlpO-recombinase-dependent step that switches off Cre and FlpO expression and simultaneously activates expression of a fluorescent reporter. We have confirmed this new mouse line works reliably with all CreERT2 lines and the 13 floxed genes tested. Given its characteristics, the *iSuRe-HadCre* allele will be an essential tool for laboratories performing conditional gene function analyses, and particularly those interested in performing single cell genetic studies or epistasis analysis.

## Materials and methods

### Animal experimentation

Mice containing the following genetic alleles were used: Tg(Myh11-CreERT2)^1Soff^ (9); Tg(Cdh5-CreERT2)^1Rha^ (10); Tg(UBC-cre/ERT2)^1Ejb^ (11); Tg(Prox1-CreERT2)^aTmak^ (12); Tg(iSuRe-Cre) (7); Tg(Tie2-Cre) (13); Tg(Sox2-cre) (14); Gt(ROSA)26Sor^tm1(EYFP)Cos^ (here called Rosa26-Lox-Stop-Lox-EYFP) (15); Gt(ROSA)26Sor^tm4(ACTB-tdTomato,-EGFP)Luo^ (here called Rosa26-Lox-MbTomato-Lox-MbEGFP) (16); Gt(ROSA)26Sor^tm14(CAG-tdTomato)Hze^ (here called Rosa26-Lox-Stop-Lox-TdTomato Ai14) (17); Gt(Rosa)26Sor^tm1(iChr2-Control-Mosaic)Ben^ (here called Rosa26-LSL-iChr2) (31); Gt(Rosa)26-LSL-Pi3KCA^H1047R^ (18); Pik3ca^tm1.1Waph^ (19); Notch1^flox^ (20); Notch2^flox^ (21); Rbpj^flox^ (22); Foxo1/3/4^flox^ (23); Vegfr2^flox^ (24,25); Flt1^flox^ (26); Dll4^flox^ (27); Jag1^flox^ (28); Myc^flox^ (29); Mycn^flox^ (30). The iSuRe-CrePEST^v1^, Rosa26-iSuRe-CrePEST^v2^, and iSuRe-HadCre alleles were produced for this study by CRISPR/Cas9 induced homologous-dependent DNA repair in the iSuRe-Cre or ROSA26 locus with the constructs indicated in the main figures. Notch3^flox^ mice were generated using guide RNAs (gctgtgttttagtatgtagt and ttgagcgttagaaagattgg) and 147bp donor oligos containing loxP sites. To activate recombination in animals containing CreERT2 alleles, 4-OH-tamoxifen (H6278) or tamoxifen (Sigma, P5648) was administered by intravenous injection to pregnant females, pups, or non-pregnant adults at the indicated stages and doses. Genotyping primers are listed in Supplementary Table S1.

All mouse husbandry and experimentation was conducted using protocols approved by local animal ethics committees and authorities (Comunidad Autónoma de Madrid CAM-PROEX 167/17, 130/19 and 164.8/20). The CNIC mouse colony (Mus musculus) is maintained in racks with individual ventilation cages according to current Spanish and European legislation (RD 53/2013 and EU Directive 63/2010, respectively). Mice have a dust- and pathogen-free bedding, sufficient nesting, and environmental enrichment material for the development of species-specific behavior. All mice have access to food and water ‘ad libitum’ in environmental conditions of 45–65% relative humidity, temperatures of 21–24 °C, and a 12 h/12 h light/dark cycle. In addition, animal welfare is preserved through an animal health surveillance program that follows FELASA recommendations for specific pathogen-free facilities. We used Mus musculus with the C57BL6, C57BL6x129SV or C57BL6xDBA2 genetic backgrounds.

### DNA constructs, engineering and genome targeting

The basic elements of the different DNA constructs depicted in the figures were obtained from Addgene or by Gene Synthesis and assembled by standard DNA cloning methods. Most sequences were obtained from DNA constructs previously generated in our lab (7,31,32). To obtain the gene targeting vector to produce the *iSuRe-HadCre* allele, we modified an existing plasmid from Addgene (#22799). New sequences were obtained by direct Miniprep DNA or PCR-based cloning with restriction enzymes. The unique restriction sites and the sequential modular cloning strategy were designed and selected using DNAstar SeqBuilder (Lasergene) in concert with our previous experience with commercially available restriction enzymes and PCR kits. The basic elements of the DNA constructs are depicted in several figures with the following abbreviations: ROSA26 (DNA sequences of the mouse ROSA26 locus located in chromosome 6), CAG prom (strong CMV enhancer + B-actin promoter), INS (Insulator), FRT (short DNA sequences recognized by the recombinase Flp or codon-optimized FlpO), Lox (short DNA sequences recognized by the recombinase Cre, LoxP, or LoxN variants, which are mutually incompatible), Int-Cre (Cre-containing intron), ERT2 (estrogen receptor domain that when fused to Cre is inducible/activable by tamoxifen), 2A (2A peptide sequence of the *Thosea asigna virus* (TaV) used for equimolar expression/translation of upstream and downstream proteins), Int-FlpO-Stop (intron containing FlpO followed by a stop codon), PEST (the Odc1 protein domain, which signals proteins for degradation), H2B (histone 2B, used in fusions to provide for chromatin localization), V5 (unique epitope for immunodetection), WPRE (woodchuck hepatitis virus element that enhances RNA stability and transport, enhancing transgene expression), pA (polyA transcription stop signal), N-PhiM (gene encoding a non-fluorescent protein that is often used as a reporter/marker of promoter expression in the absence of recombination), MbTomato (gene encoding a membrane-tagged tdTomato fluorescent protein that emits in the red spectrum when excited by a yellow laser), and PGK-Neo (selection marker previously used in embryonic stem cell gene targeting approaches).

*iSuRe-CrePEST^V1^* donor DNA was injected together with guide RNA (sequence AATCCAGAGGTTGATTAGCG) into mouse eggs collected from an intercross between male *iSuRe-Cre* and female DBA/Bl6 mice. The *iSuRe-CrePEST^V2^* and *iSuRe-HadCre* donor DNAs were injected into mouse eggs together with the guide RNA GCAACACGATCCCGCCACCA. All donor-DNA-plus-guide-RNA injections included standard CRISPR Alt RNA and Cas9 protein (IdTDNA). Several founders were obtained from these injections. After breeding to the C57Bl6 background, the progeny were PCR screened and then crossed with CreERT2 or other mouse lines to confirm proper gene targeting and the expression or function of distinct construct elements.

### Immunostaining

For immunostaining of mouse retinas, eyes were dissected from mouse pups and fixed by incubation with agitation for 20 min in 4% PFA in PBS (diluted from a stock of 16% PFA; EMS 15710). After two washes in PBS, retinas were microdissected from the eyes and refixed in 4% PFA for 45 min with agitation.

For rabbit anti-Myc immunostaining, eyes were fixed only 1 h on ice in PFA4% and retinas microdissected.

Dissected retinas were blocked and permeabilized by incubation for 1 h in PBST (0.3% Triton X-100, 3% fetal bovine serum (FBS) and 3% donkey serum in PBS). Samples were then washed twice in PBST and incubated with primary antibodies (see Supplementary Table S3) diluted in PBST overnight at 4°C with agitation. Retinas were then washed 5 × 20 min in PBST diluted 1:2. Samples were then incubated in this same solution for 2 h at room temperature with Alexa-conjugated secondary antibodies. After 3 × 15 min washes in PBS containing 0.15% Triton X-100, retinas were washed 2 × 15 min in PBS and mounted in Fluoromount-G (SouthernBiotech). For combining rabbit anti-ERG-647 with rabbit anti-Myc immunostaining, retinas were incubated with 5% rabbit serum for 30 min at r/t after the Alexa 594 AffiniPure Fab Fragment Donkey Anti-Rabbit secondary antibody, and then incubated with rabbit anti-ERG-Alexa-647 diluted in 0.15% Triton, 1.5% BSA, 1.5% Rabbit serum overnight at 4ºC, followed by washes and mounting as described above.

For immunostaining of organs cryosections, tissues were fixed for 2 h in 4% PFA in PBS at 4 °C. After three washes in PBS for 10 min each, organs were stored overnight in 30% sucrose (Sigma) in PBS. Organs were then embedded in OCT (Sakura) and frozen at − 80 °C. Cryosections (15 μm) were cut on a cryostat (Leica), washed three times for 10 min each in PBS, and blocked and permeabilized in PBS containing 10% donkey serum (Millipore), 10% fetal bovine serum (FBS) and 1% Triton X-100. Primary antibodies were diluted in blocking/permeabilization buffer and incubated overnight at 4 °C. This step was followed by three 10-min washes in PBS and incubation for 2 h with conjugated secondary antibodies and 4,6-diamidino-2-phenylindole (DAPI) in PBS at room temperature. After three washes in PBS, sections were mounted with Fluoromount-G (SouthernBiotech). All antibodies used are listed in Supplementary Table S3.

For adult aortas paraffin sections immunostaining with antibodies detecting RBPJ, aortas were fixed in 4% PFA in PBS at 4 °C, overnight. After three washes in PBS for 10 min each, aortas were stored overnight in 70% ethanol and then processed and embedded in paraffin. Paraffin sections (5 μm) were cut on a microtome (Leica), In brief, sections were dewaxed and rehydrated, followed by antigen retrieval: for anti-RBPJ immunostaining, antigen retrieval was carried out in sub-boiling sodium citrate buffer (10 mM, pH 6.0) for 20 min, and slides were then allowed to cool down to room temperature for 30 min. Sections were then washed twice with PBS and then incubated for 10 min in BloxAll (Vector laboratories SP-6000) to quench endogenous peroxidase activity. Next, slides were washed twice for 5 min each in wash buffer (0.3M NaCl, 0.05M Tris–HCl pH7.5, 0.1% Tween), followed by blocking for 1 h in PBS containing 10% FBS, 0.3% Triton and 10% donkey serum. Sections were then incubated with primary antibody in antibody incubation buffer (5% donkey serum, 1% FBS in wash buffer) overnight at 4 °C. After three washes, slides were incubated at room temperature for 1h with biotin-conjugated secondary antibodies anti-rabbit-HRP secondary antibody, and, after washing, sections were incubated another hour with ABC reagent (Vector Laboratories, PK-6100). Sections were then washed and the signal was amplified using the TSA fluorescein kit (NEL701A001KT). Sections were counterstained with DAPI and mounted with Fluoromount-G (SouthernBiotech).

### Image acquisition and analysis

Whole mount tissue samples and sections were imaged at high resolution with a Leica SP8 (+ Navigator) or a Stellaris confocal microscope, using 10×–63× objectives for confocal scanning. We acquired individual fields or tiles of large areas. All images shown are representative of results obtained for each group and experiment. For each comparison, all animals were dissected and processed under exactly the same conditions. For comparisons of phenotypes or signal intensities, images were obtained using the same laser excitation and confocal scanner detection settings. Fiji/ImageJ was used to threshold, select, and quantify objects in confocal micrographs. For quantifications, we used manual and automatic ImageJ public plugins and customized Fiji macros. In general, endothelial cell nuclei (ERG+) were segmented via the Threshold/Find maxima functions. For each segmented nucleus (each ROI), we determined reporter (MbTomato or YFP) and marker (p21 or Ki67 or Myc or Rbpj) positivity and defined new ROIs with different assigned objects. Positivity was assigned when the overall ROI pixel intensity was above a defined threshold. Counts for individual cellular reporter–marker combinations were automatically obtained and then confirmed by visual inspection of each segmented ROI. Data were copied and further analyzed in R, Excel or Graphpad.

### Isolation of lung fibroblasts from adult mice

To establish primary cell cultures of lung fibroblasts we followed a published protocol (33), with some modifications. Briefly, lungs were dissected from mice under sterile conditions and placed in sterile PBS. In a cell culture hood, lungs were removed from PBS and then chopped into small fragments with scissors and placed in 10 ml of digestion buffer containing DPBS (Thermofisher 14040141) with Liberase TL (0.14 Wunsch units/ml; Merck 5401020001) and 1× antibiotic/antimycotic (Thermofisher 15240096). Tissue in digestion buffer was incubated in a water bath at 37ºC for 30min, with mixing every 2–3 min. After this period, the solution was pipetted up and down to break clumps and after 10 ml of culture medium containing DMEM/F12 (Thermofisher 11320033), 15% FBS and 2× antibiotic/antimycotic solution was added. This cell suspension was centrifuged at 500g for 5 min. The cell pellet was washed two times with culture medium to remove liberase completely. At the end cells were resuspended in 12mL of culture medium and seeded in a p100 dish and incubated at 37ºC, 5% CO_2_, 3% O_2_. Medium was changed after 3–5 days, when fibroblasts had crawled out of tissue fragments. Fibroblasts were then expanded and maintained in full medium at 37ºC, 5% CO2, 3% O_2_ for expansion and only transferred to normoxic conditions to carry out experiments.

To induce recombination, cells were trypsinized, resuspended and plated in culture medium containing 0.2 micromolar of 4-OH tamoxifen, for 4 h, and washed after in culture medium. Cells were then trypsinized at different timepoints after induction (24, 48, 72, 96 and 120 h), stained with DAPI and analysed or sorted in a FACS machine. A minimum of 20000 cells per experimental group were sorted into 300 μl of a solution containing DPBS and 10% FBS. At the end, cells were centrifuged at 500g, for 5min at 4ºC, and supernatants removed. Cell pellets were stored at –80ºC until all samples were ready to be further processed. Genomic DNA was extracted from cells pellets by incubating each pellet in 40 μl of DirectPCR Lysis Reagent (Viagen 301-C) supplemented with proteinase K (0.33 mg/ml) and incubated at 55ºC overnight. Proteinase K was inactivated by incubating samples at 85ºC for 45 min. 1 μl of each sample was directly used for quantitative real time PCR using Taqman universal master mix and an Applied Biosystems QuantStudio5 machine.

### Derivation of mouse embryonic stem cells

To derive genetically modified mouse ES cells, we intercrossed mice containing the desired alleles (*iSure-HadCre*, with or without *UBC-CreERT2* and *Cdh5-CreERT2*), and the CNIC pluripotent cells unit expanded *in vitro* their blastocysts according to established protocols (34). Briefly, blastocysts were transferred individually to a 24 well plate containing feeder layers of freshly inactivated MEFs and in ES-2i medium (DMEM/Glutamax, GIBCO 31966–021; NEAA, ß-mercaptoethanol, LIF, 20% Serum Replacement, 3 μM CHIR and 1 μM PD). Blastocysts were cultured without disturbance for 3 days. From day 4 the medium was changed every other day, and when each ICM had grown sufficiently, was disaggregated by gently trypsinization and individually passaged to a new 24-well plate. Following several passages independent cell lines were genotyped, expanded and frozen. Some of the ES cell clones containing the desired alleles were treated with 4-OHT (concentration ranging from 0.01 to 1 μM) before or after differentiating them to endothelial cells for 6 days on a OP9 feeder monolayer in basal alpha MEM Media.

### Flow cytometry and fluorescence-activated cell sorting

**Figure 1.**
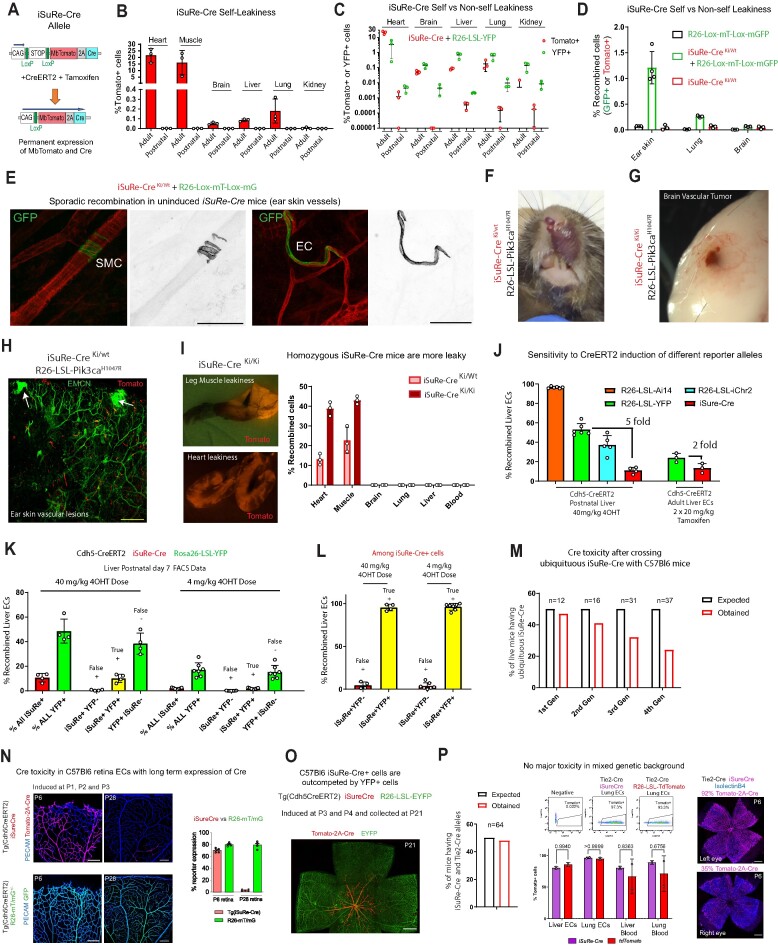
Caveats of the *iSuRe-Cre* allele. (**A**) Simplified schematic of the published iSuRe-Cre allele. After combining it with any other CreERT2 allele, is possible to induce its recombination with tamoxifen and analyse cells with permanent expression of the reporter MbTomato and Cre. (**B**) FACS analysis of *iSuRe-Cre* self-leakiness in organs from adult (8 weeks old) and postnatal mice (postnatal day 7). Note that major leakiness occurs only in adult mice. (**C**) FACS analysis of *iSuRe-Cre* self-leakiness (MbTomato^+^ cells) versus non-self-leakiness with the *Rosa26-Lox-Stop-Lox-EYFP* allele (YFP^+^ cells). In general, non-self-leakiness is higher than self-leakiness. (**D**) FACS analysis of *iSuRe-Cre* self-leakiness (MbTomato^+^ cells in red bar) versus non-self-leakiness with the *Rosa26-Lox-MbTomato-Lox-MbEGFP* allele (GFP^+^ cells in animals with or without iSuRe-Cre). (**E**) Confocal micrographs of adult mouse tissues, showing inadvertent recombination in a smooth muscle cell (SMC) and EC. (**F-H)** Images of vascular lesions on snout (**F**), brain (**G**) and ear skin (**H**) of uninduced adult *iSuRe-Cre Rosa26-Lox-Stop-Lox-Pik3ca^H1047R^* mice. (**I**) Stereomicroscopy images of leg muscle and heart tissue showing endogenous MbTomato expression due to self-leakiness in *iSuRe-Cre* mice, higher in animals with 2 copies *(ki/ki)* of the allele. (**J**) FACS analysis of the sensitivity of the indicated alleles to CreERT2-dependent recombination. (**K**) FACS analysis of the frequency of false positives (MbTomato or iSuRe+,YFP^–^), true positives (iSuRe^+^,YFP^+^), and false negatives (YFP^+^, iSuRe^–^) in liver ECs after the administration of different tamoxifen doses to mice carrying the indicated alleles. (**L**) FACS analysis of the frequency of false and true positives among *iSuRe-Cre/Tomato^+^* cells in postnatal day 7 liver ECs. (**M**) Expected and obtained survival ratios of animals containing the germline recombined *iSuRe-Cre* allele (originally on the G4 or C57Bl6x129 background) across generations of breeding with the C57Bl6 strain. (**N**) Confocal micrographs used to compare the frequency of cells positive for MbTomato-2A-Cre+ (from the *iSuRe-Cre* allele) and for GFP+ (from the *Rosa26-Lox-mT-Lox-mG* allele) in retinas of animals in the C57Bl6 background. The drop in the frequency of MbTomato-2A-Cre^+^ cells from P6 to P28 indicates Cre toxicity in ECs during the development of retinal vessels. (**O**) Representative confocal micrograph showing that when animals containing the three indicated alleles are in the C57Bl6 background, MbTomato/iSure-Cre^+^ cells are outcompeted by EYFP^+^ cells during retinal vascular development. (**P**) Expected and obtained survival ratios for mice containing the *iSuRe-Cre* and *Tie2-Cre* alleles in the mixed C57Bl6 x CD1 genetic background. These mice have similar frequencies of MbTomato^+^ blood and ECs (iSuRe-Cre + or R26-LSL-TdTomato^+^), suggesting no toxicity in this case. Confocal micrographs to the right show representative variable recombination and expression in two retinas of the same animal, with no major differences in angiogenesis (ECs are isolectinB4^+^). Data are presented as mean values ± s.d. For statistics, see Source Data File 1. Scale bars in E, 50μm; in N 200 μm; and in H, O and P, 500 μm.

With the exception of the FACS data presented in Figure 1D (see below), mouse organs were minced and digested to a single-cell suspension by incubation at 37°C for 30 min with 2.5 mg/ml type I collagenase (Thermofisher), 2.5 mg/ml dispase II (Thermofisher) and 50 ng/ml DNAseI (Roche). Cells were resuspended in 5–10 volumes of FACS buffer (PBS lacking Ca^2+^ or Mg^2+^ and containing 10% dialyzed FBS (Termofisher)) and filtered through a 70 μm filter to remove non-dissociated tissue. Erythroid cells were removed by incubating cell suspensions in blood lysis buffer (0.15 M NH_4_Cl, 0.01 M KHCO_3_ and 0.01 M EDTA in distilled water) for 10 min on ice. Cells were washed in FACS buffer, spun at 400g and resuspended in the required volume of buffer for direct FACS analysis (300 μl) or immunostaining (100–200 μl).

For the analysis of endothelial cells (CD31+, CD45–), cells were incubated at 4°C for 30 min with APC-conjugated rat anti-mouse CD31 (1:200, BD Pharmingen, 551262) and rat anti-mouse CD45-APC-Cy7 (1:200, BD Pharmingen 557659). DAPI (1:1000) was added prior to cell analysis. For the analysis or isolation of cells from dissociated tissues, viable cells were selected by the absence of DAPI fluorescence. All viable cells were interrogated by examining FSC and SSC to select by size and complexity and by comparing FSC-H and FSC-W repeated with SSC-H and SSC-W in order to discern single cells. An additional channel lacking any endogenous or fluorescent label was acquired to detect and exclude autofluorescence. Cells were selected according to their absence of DAPI and the intensity of APC, APC-Cy7, endogenous EYFP and endogenous MbTomato signals. Flow cytometry analysis and FACS were performed with BD Fortessa or BD Aria Cell Sorter. Experimental data were analyzed using Flow JO 10.5.0 or BD FACS DIVA v8.0.1 software.

For the analysis presented in Figure 1D, skin, lung, and brain samples were dissociated as follows:

For ear skin, sacrificed adult mice were perfused with PBS for 5 min to wash out erythrocytes from tissues. Ear skin was isolated, minced with dissection scissors, and digested in PBS containing 0.2 mg/ml DNase I (Roche), 0.2% FBS (Gibco™), and 5mg/ml collagenase II (Sigma) for 10 min at 37°C with shaking at 950 rpm. Digestion was stopped by adding 10 μl 0.5M EDTA, and samples were filtered through a 50 μm CellTrics filter (Sysmex). The filter was washed twice with FACS buffer (PBS, 0.5% FBS, 2 mM EDTA), after which cells were pelleted and resuspended in FACS buffer. Dead cells were labeled with sytox blue dead cell stain (Life Technologies), and GFP^+^ cells were passed on a BD LSRFortessa^TM^ Cell Analyzer (BD Biosciences). Raw data were analyzed with FlowJo software version 10.5.0 (TreeStar).

For the isolation of lung tissue, adult mice were perfused via the right ventricle with PBS for 5 min to wash out erythrocytes from the lungs. The thorax was opened, and the lungs were inflated via the trachea with a 1 ml solution of PBS containing 10 mg/ml dispase II (Sigma) and left to incubate for 5–10 min. The lungs were then removed, placed in a clean petri dish, and injected with 1.0 mg/ml collagenase II (Sigma) and 0.2 mg/ml DNase I (Roche) in PBS and incubated for 5 min. Lungs were transferred to tubes containing 1.5 ml of 1 mg/ml collagenase II and 0.2 mg/ml DNase I in PBS and minced into small pieces using scissors. Tissue samples were further incubated at 37°C with shaking at 750 rpm for 10 min. Samples were then sheared by repeated pipetting with a 1000 μl pipette and incubated for an additional 5 min. Digestion was quenched by addition of EDTA to a final concentration of 2 mM. The resulting cell suspensions were then filtered through 50 μm CellTrics filters (Sysmex). Filters were washed twice with FACS buffer (PBS, 0.5% FBS, 2 mM EDTA), after which cells were pelleted and resuspended in FACS buffer. Dead cells were labeled with sytox blue dead cell stain, and GFP^+^ cells were passed on a BD LSRFortessa™ Cell Analyzer. Raw data were analyzed with FlowJo software version 10.5.0.

For the dissociation of adult brain tissue, sacrificed mice were perfused with PBS for 5 min to wash out erythrocytes. Brains were digested to a single cell suspension using the Neural Tissue Dissociation Kit (P) (Miltenyi Biotec). Briefly, the brain was removed from the skull and minced with scissors until no large pieces remained. Minced tissue was transferred to a tube containing 5 ml of papain-based enzyme mix and incubated for 17 min at 37°C with slow rotation. Enzyme mix 2 was added, and the tissue was sheared by repeated pipetting with a 1000 μl pipette and incubated for an additional 12 min. The tissue was then sheared further by passing through a 20 G syringe needle and incubated for another 10 min. The resulting cell suspension was further diluted by addition of 10 ml cold PBS and passed through a nylon 70 μm cell mesh (Corning). Cells were pelleted and resuspended in 500 μl FACS buffer, and GFP^+^ cells were immediately passed on a BD LSRFortessa™ Cell Analyzer.

### DNA profiling of FACS-sorted cells

Cells were suspended in PBS with no Ca^2+^ or Mg^2+^ and containing 10% dialyzed FBS (Termofisher) and were sorted according to YFP, MbTomato, anti-CD31-APC or anti-CD45-APC-Cy7 fluorescent signals.

For DNA isolation, between 5 and 40 thousand cells were obtained per sample. Cells were sorted at a slow flow rate (high purity scale) and with a 100 μm nozzle. Cells were pelleted by spinning at 500g for 5 min and resuspended in 25 μl DirectPCR (Cell) Lysis Reagent for PCR (VIAGEN Cat #301-C) containing a final concentration of 0.4 mg/ml proteinase K. After incubation at 55°C overnight, the proteinase was inactivated at 85°C for 45 min. Semi-quantitative and competitive PCR (Supplementary Figure S6) was performed with 1μl of DNA per sample using the primers listed in Supplementary Table S1. Groups of 2–4 primers were used per PCR reaction. Some of the PCRs served as DNA input controls (for semi-quantitative PCR), and others targeted the different floxed gene sequences.

To quantify the amount of the different *iSuRe-HadCre* allele DNA elements before and after recombination (Supplementary Figure S4A), we used the online software from Integrated DNA technologies (IdT) to design the probe and primer pairs (listed in Supplementary Table S2). Quantitative real-time PCR was performed with 1μl of DNA using Taqman universal master mix (Applied Biosystems 4304437) and an Applied Biosystems QuantStudio5 machine.

### Single cell RNAseq analysis

RNA-seq data can be viewed at the Gene Expression Omnibus (GEO) under accession number GSE245726. Instructions and code to reproduce all scRNA-seq results can be found at https://doi.org/10.5281/zenodo.11220492. For scRNA-seq data processing the following pipeline was followed. For alignment and quantification of gene expression the reference transcriptome was built using mouse genome GRCm38 and ensembl gene build version 98 (sep2019.archive.ensembl.org). The Cre, sv40pA, Tomato or WPRE transgenes sequences expressed in the samples were added to the reference. Gene meta-data were obtained from the corresponding Ensembl BioMart archive. Reads from hashtags and transcripts were processed, aligned, and quantified using the CellRanger v7.1.0 pipeline. Single cell analysis was based on the Seurat R^50^ package. Low quality cells were filtered out using the following criteria: total counts >2000 and below 75 000, genes detected >500 and below, Mitochondrial transcripts content <5%, Haemoglobin transcripts <5%, Hashtag counts >4 applying a 0.99 positive quantile threshold with the function HTODemux, where doublets were filtered out. Cells were de-multiplexed using the sample hashtag antibody signals (Biolegend). Counts were log-normalized and cells expressing normalized counts of Cdh5 ≤0.9 and WPRE ≤0.5 were removed to select Tomato + expressing ECs for the analysis and remove other contaminant cell types.

### Statistical analysis

All bar graphs show mean ± s.d. Experiments were repeated with independent animals. Comparisons between two groups of samples with a Gaussian distribution were by unpaired two-tailed Student *t*-test. Comparisons among more than two groups were made by one or two-way analysis of variance (ANOVA) followed by multiple comparison tests as indicated in Source Data File 1. All calculations were done in Excel, and final datapoints were analyzed and represented with GraphPad Prism. No randomization or blinding was used, and animals or tissues were selected for analysis based on their genotype, the detected Cre-dependent recombination frequency, and the quality of multiplex immunostaining. Sample sizes were chosen according to the observed statistical variation and published protocols.

## Results

### Caveats with the first-generation *iSuRe-Cre* allele

The first generation *iSuRe-Cre* allele is a transgene located in chromosome 17. Once combined with a *CreERT2* expressing allele, recombination can be induced with the ligand tamoxifen, which leads to the permanent equimolar expression of the reporter MbTomato and Cre (Figure 1A). This allele ensures high Cre activity and deletion of floxed genes in cells expressing the reporter, increasing the efficiency of conditional genetics in these cells. However, and as reported previously, and detailed further below, there are a number of caveats with the first generation *iSuRe-Cre* technology.

The first is the inadvertent recombination of the allele in a large fraction of adult myocytes in heart and muscle, a phenomenon we call self-leakiness (Figure 1B). This self-leakiness arises in the absence of any exogenous Cre or CreERT2 activity and is likely caused by skipping of the transcription stop signals and residual expression of the downstream *MbTomato-2A-Cre* cassette. This residual expression is likely to occur in cells in which the upstream CAG promoter is strongly transcribed (CMV enhancer element + beta-actin promoter). Myocytes highly express this promoter because of their continuously high production of actin, and this appears to facilitate *iSuRe-Cre* self-recombination in these cells as animals age (7). More recently, we observed the phenomenon of non-self-leakiness in other cell types. In this case, the very low level of leaky *MbTomato-2A-Cre* expression is insufficient to self-recombine the *iSuRe-Cre* allele, but is enough to recombine other floxed alleles that are more sensitive to Cre/CreERT2 activity, particularly in adult mice (Figure 1C–E). In addition, when this non-self-leakiness confers a competitive proliferative advantage to the sporadically recombined cells, for instance when using *iSuRe-Cre* in conjunction with the *Rosa26-LSL-Pik3ca^H1047R^* allele, it leads to the development of inadvertent *Pik3ca^H1047R^*-driven vascular malformations (Figure 1F-H and Supplementary Figure S1A). As expected, self-leakiness and non-self-leakiness are both increased in animals harboring 2 copies of the *iSuRe-Cre* allele (Figure 1I).

The second caveat is the relatively low sensitivity of the *iSuRe-Cre* allele to Cre/CreERT2 activity compared with other *Rosa26*-based reporter lines (Figure 1J), which increases the frequency of cells having recombination of other floxed alleles but missing recombination of the *iSuRe-Cre* allele, named here as false negatives (Figure 1K and Supplementary Figure S1A). This relatively low sensitivity to recombination may be because of the DNA structure or genomic location of the *iSuRe-Cre* allele.

A third potential caveat with the first generation *iSuRe-Cre* line is the permanent expression of Cre in reporter-expressing cells. Although this feature is what ensures full genetic deletions and significantly decreases the frequency of false positives (Figure 1K, L), it also has the potential to generate cell toxicity. Particularly *in vitro*, Cre is toxic when expressed at high levels or for long periods, presumably due to recombination of native DNA sequences resembling LoxP sites (35–38). We detected no overt Cre toxicity in cells of *iSuRe-Cre* mice when it was initially generated on the 129Sv/C57Bl6 background (7). However, after two generations of breeding to the reference C57Bl6 mouse strain, we began to notice a decrease in the percentage of germline recombined *iSuRe-Cre* mutants among newborns (Figure 1M). We also obtained evidence that when the allele was induced in C57Bl6 retinal ECs between postnatal days 1 (P1) and P3, a large fraction of the iSuRe-Cre^+^ cells seen at P6 could not be detected at P28, a phenomenon not seen with other standard Cre-reporters (Figure 1N). Most of the MbTomato-2A-Cre^+^ cells were outcompeted during vascular development and did not contribute to the capillary network, instead contributing only to more mature vessels (Figure 1O). This could reflect a decrease in cell fitness or toxicity resulting from the continuously high Cre expression. FACS analysis revealed that *iSuRe-Cre* expression was 4–8 times higher in postnatal angiogenic retinal and brain ECs than in other ECs (Supplementary Figure S1B), which may aggravate this issue specifically in these cells. However, in most of our experiments with mice on other mixed genetic backgrounds (129SvxC57Bl6 or CD1) or at adult stages, we did not detect Cre-toxicity, even when *iSuRe-Cre* was combined with additional Cre expressing alleles and a very high percentage of cells expressed the *iSuRe-Cre* allele (Figure 1P and Supplementary Figure S1C).

Since the expression of transgenes, including the synthetic CAG promoter used in the *iSuRe-Cre* allele, varies according to the cell type, organ, stage of tissue development (Supplementary Figure S1B), and the mouse genetic background (39), with this allele it is not possible to ensure the absence of Cre toxicity in all cell types, developmental stages or mouse strains.

### Design and characterization of *iSuRe-CrePEST* alleles

**Figure 2.**
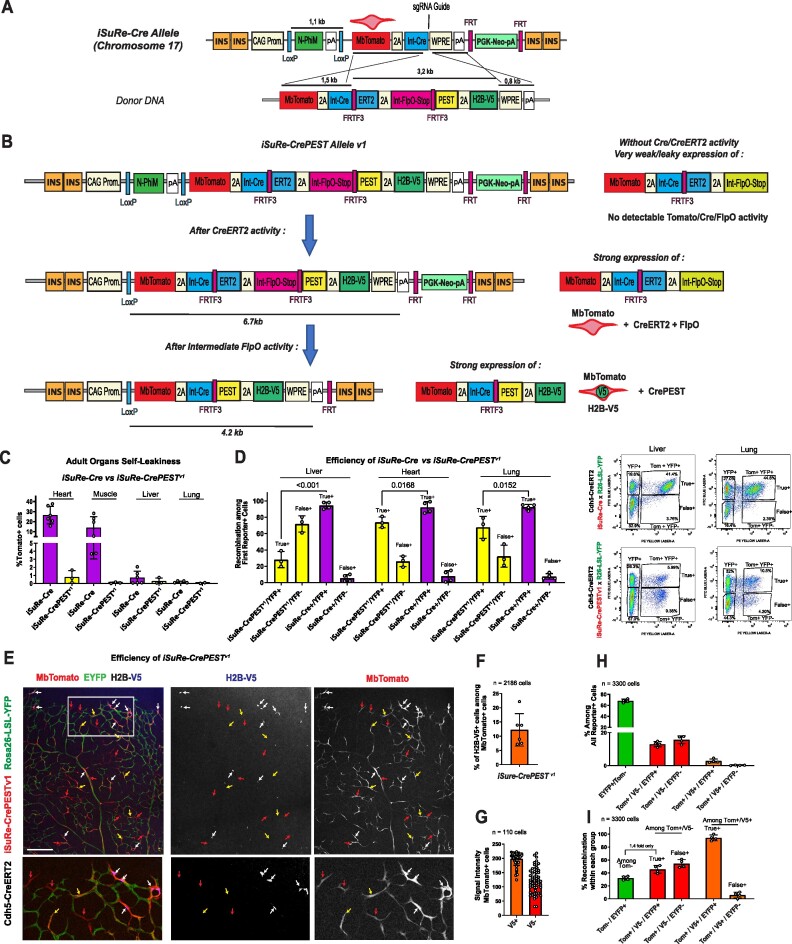
Characterization of the *iSuRe-CrePEST^v1^* allele. (**A**) Crispr-Cas9 genetic targeting of the preexisting *iSuRe-Cre* allele, present in Chromosome 17, with the indicated donor DNA to generate the new *iSuRe-CrePEST^v1^* allele shown below. (**B**) Genetic cascade and expression outcomes after induction of the *iSuRe-CrePEST^v1^* allele with CreERT2 + tamoxifen. After the first recombination event induced by the activation of CreERT2 with tamoxifen, cells will express MbTomato, CreERT2 and FlpO. In a second step, MbTomato^+^ cells with high FlpO activity will undergo genetic fusion of the PEST domain to Cre and co-express equimolar amounts of MbTomato, CrePEST, and H2B-V5. CrePEST activity is higher than CreERT2 but, due to its higher degradation, presumably lower than Cre activity. Other abbreviations and construct elements are detailed in the DNA engineering section in Methods. (**C**) FACS analysis of MbTomato^+^ cells in *iSuRe-Cre* and *iSuRe-CrePEST^v1^* animals reveals significantly less self-leakiness in the latter. (**D**) FACS analysis and charts showing the frequency of false positives (MbTomato^+^, YFP^–^) and true positives (MbTomato^+^, YFP^+^) among MbTomato^+^ cells from several adult organs of animals containing the *Cdh5-CreERT2, iSuRe-CrePEST^v1^* and *Rosa26-LSL-YFP* alleles (yellow bars) or the *Cdh5-CreERT2, iSuRe-Cre*, and *Rosa26-LSL-YFP* alleles (magenta bars). Recombination efficiency (true positives frequency) is lower in mice containing the *iSuRe-CrePEST^v1^* allele. (**E**) P6 retina confocal micrographs showing the efficiency of the recombination cascade (MbTomato activation followed by H2B-V5) of the *iSuRe-CrePEST^v1^* allele (induced with Cdh5-CreERT2 and tamoxifen at P1). Red arrows indicate cells with only MbTomato expression (false positives); yellow arrows, cells with MbTomato and EYFP expression (partial recombination of the *iSuRe-CrePEST^v1^* allele); and white arrows, cells with expression of MbTomato, EYFP, and H2B-V5 (true positives). (**F**) Chart showing the low efficiency (% of V5+ cells) of the second, FlpO-mediated, *iSuRe-CrePEST^v1^* recombination event in MbTomato+ cells (these were initially induced by CreERT2 at P1). (**G**) Quantification of MbTomato reporter signal intensity (from confocal images) in cells that have undergone only the first recombination event (H2B-V5^–^) or all recombination events (H2B-V5^+^). Each dot is one cell intensity. (**H**) Frequency of cells expressing the different reporters combinations (EYFP, MbTomato, and H2B-V5) among all recombined cells in P6 retinas of mice containing the alleles *iSuRe-CrePEST^v1^*, Rosa26-LSL-YFP and Cdh5-CreERT2. Note the very low frequency of MbTomato^+^, H2B-V5^+^ cells, but that most of these are YFP^+^ (true positives). (**I**) Frequency of false positives (YFP^–^) and true positives (YFP^+^) among MbTomato^–^ cells (green bar) or MbTomato^+^, H2B-V5^–^ cells (red bars) or MbTomato^+^, H2B-V5^+^ cells (orange bars), revealing that only H2B-V5^+^ cells accurately indicate cells with high Cre activity, thanks to the permanent CrePEST expression. Data are presented as mean values ± s.d. For statistics, see Source Data File 1. Scale bar, 200 μm.

We set out to improve the iSuRe-Cre technology by generating more refined genetic constructs and mouse lines. Our first attempt involved the design of a construct that would be non-leaky in all cell types and that would allow us to induce the expression of a modified Cre with weaker activity (CrePEST). CrePEST is a fusion of Cre with the PEST domain of ornithine decarboxylase (*Odc1*) to destabilize Cre, a strategy that has been used in Drosophila to bypass Cre toxicity (40,41). We first targeted the *iSuRe-CrePEST* construct to the same *iSuRe-Cre* mouse allele on chromosome 17 using CRISPR/Cas9-mediated homologous-dependent repair (Figure 2A).

To overcome leakiness, we fused Cre to ERT2, so that any skipping of the upstream transcriptional stop signal would result in the expression of CreERT2, and not Cre (compare Figure 2A  *iSuRe-Cre* to Figure 2B  *iSuRe-CrePEST^v1^*). In this way, any residual expression of CreERT2 would be trapped in the cytoplasm, which would prevent inadvertent recombination of other floxed alleles (leakiness). This strategy proved to be effective, with animals containing the *iSuRe-CrePEST^v1^* allele showing minimal leakiness in cardiomyocytes and skeletal myocytes (Figure 2C and Supplementary Figure S2A).

Recombination of the *iSuRe-CrePEST^v1^* allele by CreERT2 results in a cascade of recombination events (Figure 2B). Surprisingly, a significant fraction of MbTomato + cells did not recombine other floxed reporter alleles (and were hence false positives) even 6 days after the induction of recombination and expression of the *iSuRe-CrePEST^v1^* allele (Figure 2D and Supplementary Figure S2B). This low recombination efficiency could be due either to weak expression or activity of FlpO in the first step of the cascade (after the first Cre-ERT2 dependent LoxP recombination) or to weak CrePEST activity after FlpO recombination. Analysis of the expression of H2B-V5, a marker of cells that have undergone FlpO recombination (see Figure 2B genetic cascade), showed that only a small fraction of MbTomato + cells expressed H2B-V5 (Figure 2E, F). This demonstrates that the intermediate cascade step of FlpO-dependent recombination, necessary for full construct activation, does not occur in a significant fraction of MbTomato+ cells, even 4 days after induction with 4-OH tamoxifen (4-OHT), possibly due to relatively weak expression and activity of FlpO driven by the recombined *iSuRe-CrePEST^v1^* allele. We also noticed that whereas cells with a strong MbTomato+ signal co-expressed H2B-V5, cells weakly expressing MbTomato were H2B-V5 negative (Figure 2E, G). Importantly, the frequency of *Rosa26-Lox-Stop-Lox-EYFP* allele recombination was much higher in the few H2B-V5^+^ cells than in H2B-V5^–^ cells (Figure 2E, H, I), suggesting that CrePEST expression is sufficient to fully recombine other floxed alleles. Overall, these results show that the *iSuRe-CrePEST^v1^* allele is unreliable because it generates many false positives. The sensitivity to CreERT2 recombination of the *iSuRe-CrePEST^v1^* allele is also significantly lower than that of other Rosa*26*-based reporter alleles (Figure 2E, H).

**Figure 3.**
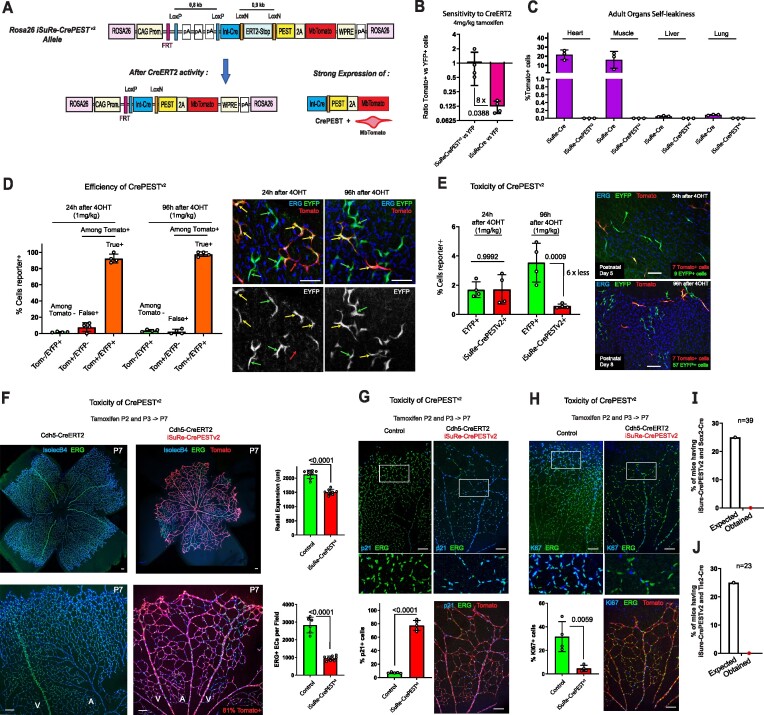
Characterization of the *Rosa26-iSuRe-CrePEST^v2^* allele. (**A**) Schematic of the *Rosa26-iSuRe-CrePESTv2* allele. Tamoxifen–induced CreERT2 activity leads to deletion of the stop cassette containing three Sv40 polyA sequence repeats, triggering co-expression of CrePEST and the reporter MbTomato. (**B**) Confocal microscopy analysis of the sensitivity to CreERT2-induced recombination of the *Rosa26-iSuRe-CrePEST^v2^* and *iSuRe-Cre* alleles (MbTomato^+^) relative to the internal control *Rosa26-LSL-EYFP* allele (each dot represents one retina sample). (**C**) FACS analysis of the self-leakiness frequency (MbTomato + cells) in adult organs of *iSuRe-Cre* versus *Rosa26-iSuRe-CrePEST^v2^* animals (each dot represents a measure in one animal). (**D**) Quantification of confocal micrographs reflecting the relative frequency of MbTomato+ cells (*Rosa26-iSuRe-CrePEST^v2^*) and YFP^+^ cells (*Rosa26-LSL-EYFP*) 24 and 96 h after tamoxifen induction at P4. Most MbTomato^+^ cells are already YFP^+^ (true positives, yellow arrows) at 24 h postinduction. Very few are MbTomato^+^ and YFP^–^ (false positives, red arrow) at 24 h, and almost none later. (**E**) MbTomato-2A-CrePEST^+^ cells become 6 times less frequent 4 days after induction, indicating toxicity of CrePEST expression. (**F–H**) Retina confocal micrographs and corresponding charts showing a significant decrease in angiogenesis and cell proliferation in animals containing the induced *Rosa26-iSuRe-CrePEST^v2^* allele. Each dot in the charts represents one large retina field (in total 15403 cells were quantified in G and 8795 in H). (**I, J**) Animals containing the *Sox2-Cre* (recombines all cells) or *Tie2-Cre* (recombines only ECs and blood) allele in combination with the *Rosa26-iSuRe-CrePEST^v2^* allele are not born. Data are presented as mean values ± s.d. For statistics, see Source Data File 1. Scale bars, 70 μm in D and 120 μm in E–H.

We previously observed that *Rosa26* reporter alleles, particularly the *Rosa26-Lox-Stop-Lox-TdTomato (Ai14)* allele (17), are more sensitive to CreERT2/tamoxifen-induced recombination than the *iSuRe-Cre* allele (Figure 1J). Since the genetic distance between LoxP sites is similar in the *iSuRe-Cre* allele (1.1kb) and the *Rosa26-Lox-Stop-Lox-TdTomato (Ai14)* allele (0.9kb), we hypothesized that the higher sensitivity of the latter to CreERT2 recombination was due either to its different genomic location or to the DNA sequences included in its floxed transcription stop cassette (LoxP-3xpA-LoxP). Therefore, we assembled a *Rosa26*-targeting construct containing exactly the same *CAG-LoxP-3xpA-LoxP* cassette followed by a conditional *Cre-LoxN-ERT2-stopCodon-LoxN-PEST-2A-MbTomato* cassette (Figure 3A). This strategy relies on the incompatibility between LoxN sites and LoxP sites, such that the native Cre sequence will still be retained after the two initiating Cre/CreERT2-dependent deletion events (between the 2 LoxP sites and the 2 LoxN sites). Analysis of the relative recombination frequency between this new *Rosa26-iSuRe-CrePEST^v2^* allele and the internal reference *Rosa26-LSL-YFP* allele revealed that it is around 8 times more sensitive to CreERT2 activity at 4 mg/kg tamoxifen than the previous *iSuRe-Cre* allele (Figure 3B), despite the presence of two independent LoxP and LoxN flanked cassettes. This higher sensitivity significantly reduces the occurrence of false negatives. We also confirmed the *Rosa26-iSuRe-CrePEST^v2^* allele is not leaky (Figure 3C).

We next determined its reliability as a reporter of other floxed genes deletion. Just 24 h after tamoxifen induction, 90% of MbTomato-2A-CrePEST+ cells already recombined the *Rosa26-Lox-Stop-Lox-EYFP* allele, reaching 99% at 96h after induction (Figure 3D).

We next examined the potential for toxicity arising from permanent CrePEST expression driven by the strong *Rosa26-CAG* promoter (Figure 3A), particularly in highly proliferative or migratory cells that we previously found to be sensitive to the high Cre levels driven by the *iSuRe-Cre* allele (Figure 1M-O). Unfortunately, expression of MbTomato-2A-CrePEST in retinal ECs for 4 days significantly decreased the frequency of these cells relative to EYFP^+^ cells (Figure 3E), indicating cumulative toxicity linked to high CrePEST expression. Consistent with this finding, induction of the *Rosa26-iSuRe-CrePEST^v2^* allele at high frequency significantly compromised retinal angiogenesis (Figure 3F). This outcome was associated with decreased proliferation (Ki67) and increased expression of the cell-cycle inhibitor and senescence marker p21 (Figure 3G, H). We also crossed these mice with C57Bl6 *Sox2-Cre* and *Tie2-Cre* mice. Permanent expression of the induced *Rosa26-iSuRe-CrePEST^v2^* allele in all cells of the mouse embryo resulted in 100% embryonic lethality, being the *Rosa26-iSuRe-CrePEST^v2^* allele more toxic than the *iSuRe-Cre* allele (compare Figure 3I and J with Figure 1M and 1P). This significantly higher toxicity of the *Rosa26-iSuRe-CrePEST^v2^* allele is likely due to its much stronger promoter activity (Supplementary Figure S3).

Taken together, these results show that the *Rosa26-iSuRe-CrePEST^v2^* allele is more sensitive to CreERT2 and is not leaky, however, the permanent and high expression of CrePEST is also toxic to mammalian cells and detrimental to tissue development.

### Design and validation of the *iSuRe-HadCre* allele

**Figure 4.**
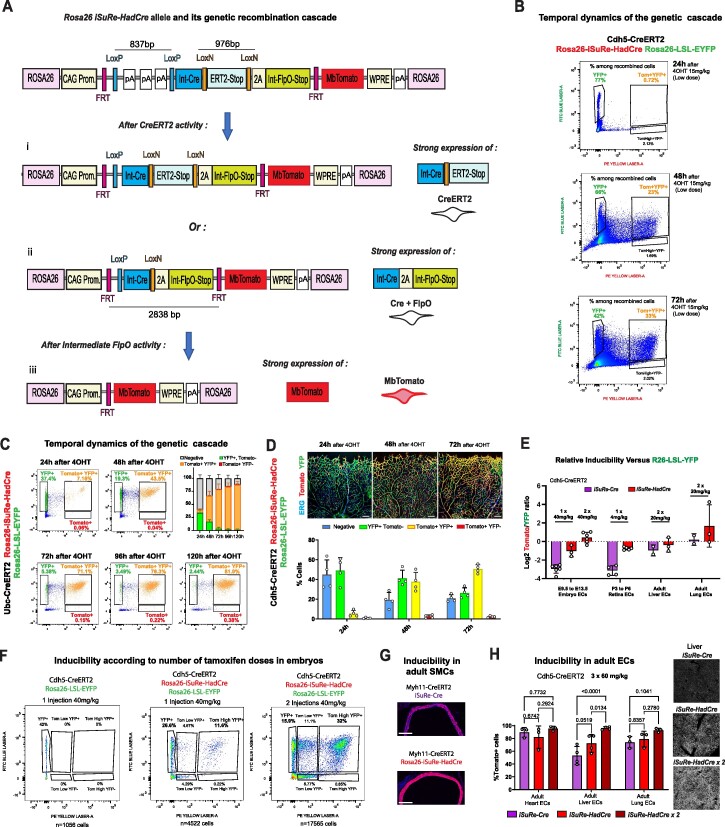
Design and validation of the *Rosa26-iSuRe-HadCre* mouse allele. (**A**) Construct and sequential genetic cascade after induction of the *Rosa26-iSuRe-HadCre* allele. After induction of CreERT2 with tamoxifen, the first recombination event results in deletion of either the LoxP or the LoxN cassette, or of both Lox-flanked genetic cassettes. Since the LoxP-flanked DNA cassette is easier to recombine than the LoxN-flanked cassette, in the presence of relatively low levels of CreERT2 induction, LoxP recombination will predominate after a single 4-OHT injection (as illustrated in the figure), and this will trigger very strong expression of CreERT2 after. This strong CreERT2 expression will facilitate/enhance LoxN cassette recombination, especially if a second injection of tamoxifen (4-OHT) is delivered after 24 h. After recombination of both LoxP and LoxN-flanked cassettes (after a single or multiple injections of tamoxifen), there will be strong, equimolar co-expression of Cre and the weaker FlpO recombinase. FlpO will recombine FRT sites, self-deleting the construct and activating expression of the reporter MbTomato in cells that had, but no longer have, high levels of Cre expression. (**B**) FACS analysis of the temporal dynamics of the genetic cascade upon 4-OHT induction. Littermate pups with the indicated alleles were injected with a single 4-OHT dose at P4, and lung cells were collected at P5, P6 or P7. (**C**) FACS analysis of the kinetics of recombination upon induction of the *Rosa26-iSuRe-HadCre* allele in fibroblasts derived from the indicated mice. (**D**) Confocal analysis of the temporal dynamics of the genetic cascade upon induction of the *Rosa26-iSuRe-HadCre* allele in retinal vessels after 15 mg/kg dose of 4-OHT. Note that the dose of 4-OHT used is low and will not recombine all the reporters in all cells. (**E**) Relative inducibility of the *iSure-Cre* and *Rosa26-iSuRe-HadCre* alleles in relation to the reference *Rosa26-LSL-EYFP* allele at different developmental stages and in different organs. (**F**) FACS analysis of the correlation between the number of tamoxifen doses and the frequency of recombination. Two doses induce higher recombination rates of the *Rosa26-iSuRe-HadCre* allele in relation to the *Rosa26-LSL-EYFP* allele. (**G**) Comparative analysis on the recombination rate in adult aorta SMCs (Myh11-CreERT2^+^). (**H**) Comparative analysis of recombination rates at very high doses of tamoxifen in adult animals, some with 2 copies (2×) of the iSuRe-HadCre allele. Each dot in the charts represents the mean value obtained in one animal. Data are presented as mean values ± s.d. For statistics, see Source Data File 1. Scale bars, 200 μm.

Given the results obtained, we surmised that we had to develop a new construct enabling high and transient Cre expression. To achieve this, we assembled the *iSuRe-HadCre* construct and targeted it to the *ROSA26* locus (Figure 4A). In mice with this allele, tamoxifen-induced CreERT2 expression triggers a sequential genetic cascade in which Cre and FlpO are first transiently expressed at equimolar levels, given the viral 2A peptide used (42), followed by an inbuilt FlpO-dependent switch-off mechanism that simultaneously self-deletes both the Cre and FlpO genes and activates expression of the reporter MbTomato (Figure 4A). We selected the FlpO recombinase and FRT sites due to their incompatibility with the Cre/CreERT2 system and their significantly lower recombination frequency in mammalian cells at 37°C (43). With this approach, we reasoned that Cre would have sufficient time to execute its action on other floxed genes before FlpO accumulated to the level needed to recombine and excise the large (2.8 kb) FRT-flanked cassette (Figure 4Aii and Aiii) from the *Rosa26-iSuRe-HadCre* allele.

To test the effectiveness of the *iSuRe-HadCre* design, we first performed a temporal FACS and histology analysis of the sequential genetic cascade, examining tissues collected at 24–120 h post-induction. The combination of the 4-OHT-CreERT2 activation and high Cre expression resulted in immediate recombination and expression of the *Rosa26-Lox-Stop-Lox-EYFP* allele, being the protein fluorescence detectable in most cells at 24–48 h postinduction (Figure 4B–D). In contrast, due to the lag in the induction of the weaker FlpO recombinase activity (43), and the subsequently slower FlpO-dependent genetic recombination, most cells expressed the iSuRe-HadCre MbTomato reporter only at 48–72 h post induction. This could be confirmed experimentally, since Cre and FlpO recombine the iSuRe-HadCre allele immediately in the first 24–48 h (Supplementary Figure S4A), while the Tomato reporter shows only at 48–72 h (Figure 4B–D). We predicted that this lag in the FlpO expression and recombination-dependent switch off activity on the iSuRe-HadCre allele, would be sufficient to express enough Cre and ensure efficient Cre-mediated deletion of other floxed genes during this time period. Indeed, >95% of MbTomato^+^ cells were also YFP^+^ at all stages and tissues analysed (Figure 4C, D), demonstrating the effectiveness of recombination in cells with transient Cre expression driven by the iSuRe-HadCre allele. Further analysis of this is presented in the next section.

We next compared the sensitivities to CreERT2 of the *iSuRe-HadCre* and *iSuRe-Cre* lines. To have an internal control, we used animals also having the *Rosa26-Lox-Stop-Lox-EYFP* allele and obtained the relative recombination data (MbTomato:YFP recombination log ratio). This analysis showed that sensitivity to CreERT2 and the relative recombination of reporter alleles vary within the same cell type (ECs) and across developmental stages and organs, being the *Rosa26-iSuRe-HadCre* allele superior to the *iSuRe-Cre* allele (Figure 4E and Supplementary Figure S4B), and in some cases also superior to that of the *Rosa26-Lox-Stop-Lox-EYFP* allele (MbTomato:YFP recombination log ratio >0 in Figure 4E), particularly when two tamoxifen doses were used. The *Rosa26-iSuRe-HadCre* allele has two floxed cassettes (flanked by the wildtype LoxP sites and the mutant LoxN sites). After recombination of the LoxP-flanked cassette, high CreERT2 expression is induced (Figure 4A, i), and this significantly enhances the recombination of the more difficult to recombine LoxN cassette (Supplementary Figure S4A) after a second tamoxifen dose (Figure 4E and F). Besides being more sensitive to *Cdh5-CreERT2* allele induction, the *Rosa26-iSuRe-HadCre* allele was also more sensitive to *Myh11-CreERT2* induction in smooth muscle cells (SMCs, Figure 4G). By using three high-dose (60 mg/kg each) tamoxifen injections, we also confirmed that the intermediate FlpO-dependent recombination is not evaded in quiescent adult tissues, which are usually more difficult to recombine (Figure 4H and Supplementary Figure S4C).

**Figure 5.**
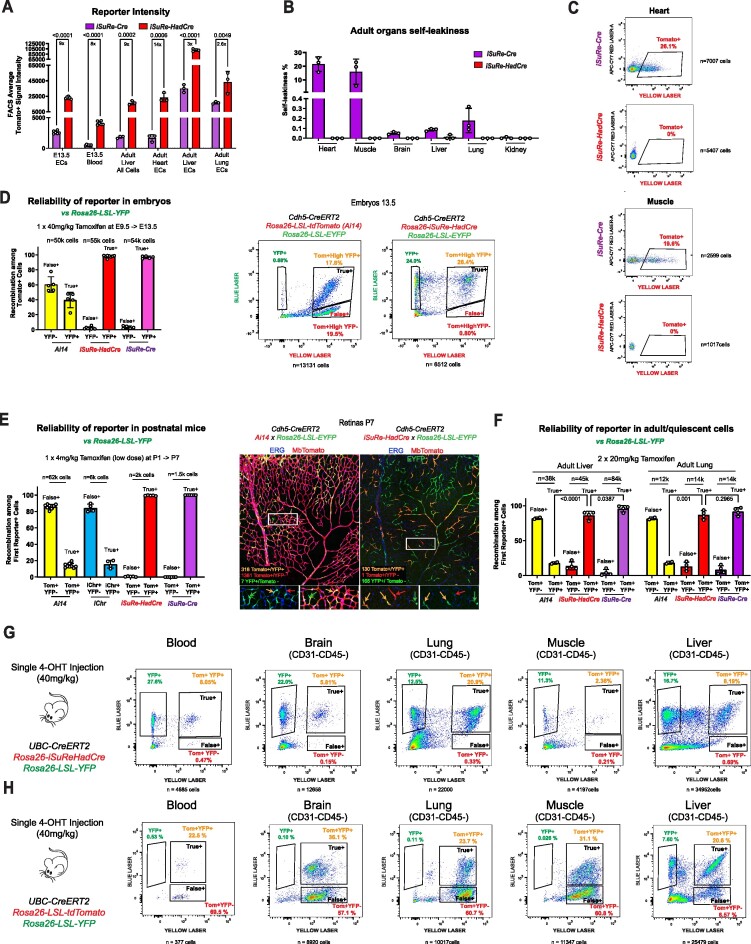
*Rosa26-* *iSuRe-HadCre* is bright, non-leaky and generates a high rate of true positives. (**A**) Comparative FACS analysis of reporter (MbTomato) intensity in recombined cells at different stages and in different organs. (**B**) Comparative FACS analysis of self-leakiness in *iSure-Cre* and *iSuRe-HadCre* mice organs. (**C**) Representative FACS plots showing no leakiness in *iSuRe-HadCre* mice organs. (**D**) Comparative FACs analysis of the rate of true positives (YFP^+^) and false positives (YFP^–^) at embryonic stages in different mouse lines when combined with the compatible reference *Rosa26-LSL-YFP* allele. (**E**) Comparative confocal analysis of the rate of true and false positives in postnatal retinas from different mouse lines when combined with the compatible reference *Rosa26-LSL-YFP* allele. (**F**) Comparative FACS analysis of the rate of true and false positives in adult liver and lung cells; recombination in these quiescent cells is generally more difficult to achieve than in embryonic or postnatal cells. (**G**, **H**) Analysis by FACS of the frequencies of YFP+ (blue laser) and MbTomato+ (yellow laser) CD31^−^CD45^−^ cells (all organ cells except CD45+ blood and CD31+ ECs) extracted from the indicated mice six days after receiving a single injection of 4-OHT. Note the difference in the frequency of false positives between the *iSuRe-HadCre* and the *Rosa26-LSL-tdTomato* line. Data are presented as mean values ± s.d. For statistics, see Source Data File 1. Scale bars, 200 μm.

In addition, we found the expression and signal intensity of the reporter expressed by the *Rosa26-iSuRe-HadCre* allele to be significantly higher which enhances its detection by direct fluorescence imaging, FACS or scRNAseq (Figure 5A and Supplementary Figure S5A, S5B). Moreover, the *Rosa26-iSuRe-HadCre* was not leaky in any cell type or organ analyzed (Figure 5B, C and Supplementary Figure S5C).

### *iSuRe-HadCre* significantly increases the efficiency of Cre-genetics in all cell types

A key feature of the *iSuRe-Cre* technology is the very high percentage of true positives (cells expressing a fluorescent reporter that also have recombination of other floxed alleles), which results from the permanent co-expression of Cre and the reporter. In contrast, reporter-expressing cells in *iSuRe-HadCre* mice express Cre only transiently. To check for the efficiency of recombination in cells expressing the *iSuRe-HadCre* allele reporter, we intercrossed several reporters and the *iSuRe-Cre* allele with the *Cdh5-CreERT2* allele and analysed embryos, postnatal and adult mice. Remarkably, the frequency of *Rosa26-Lox-Stop-Lox-EYFP* reporter allele recombination was very high in MbTomato+ cells of the *iSuRe-HadCre* line, much higher than in cells expressing other classical reporters and similar to cells permanently expressing Cre driven by *iSuRe-Cre* (Figure 5D–F). We also intercrossed the *iSuRe-HadCre* allele with the *TgUBC-CreERT2* allele (11), that is ubiquitously expressed and recombines most cell types in different organs. A single injection of 4-OHT showed that the *iSuRe-HadCre* allele also enables very efficient recombination of the *Rosa26-Lox-Stop-Lox-EYFP* reporter within MbTomato+ cells, for all cell types and organs analysed (Figure 5G and Supplementary Figure S5D). The average rate of true positives among all the organs analysed was 95%, similar to the first generation *iSuRe-Cre* (7), and significantly higher than the 20–30% obtained with standard Cre-reporters (Figure 5D–F and H, Ai14/YFP bars).

### *iSuRe-HadCre* enables complete gene deletion in single cells or whole tissues

**Figure 6.**
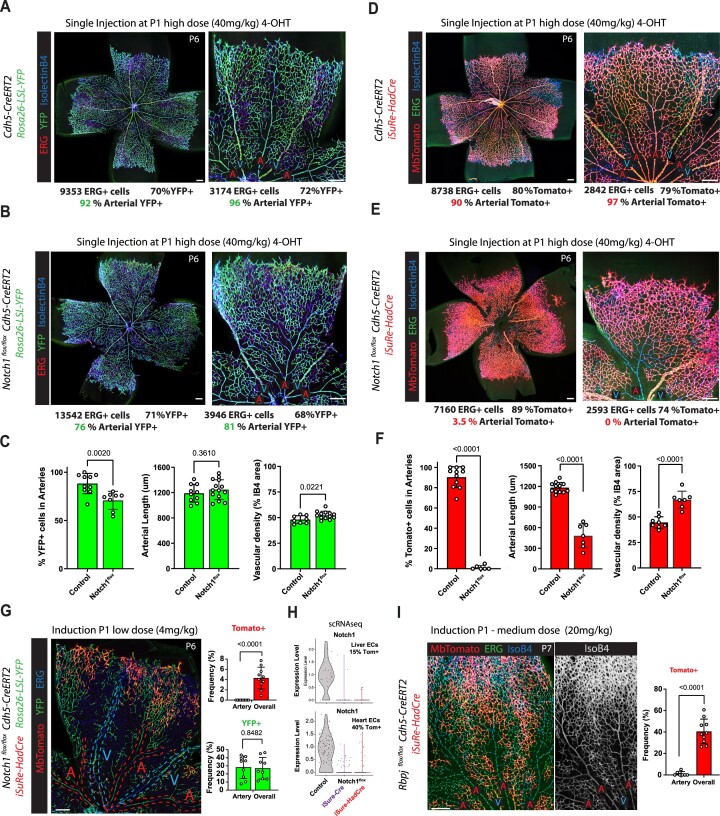
*Rosa26-* *iSuRe-HadCre* increases the efficiency and reliability of conditional genetics. (**A–C**) Representative confocal micrographs of P6 retinal vessels from control mice (**A**) and Notch1 loss-of-function (LOF) mice (**B**) with high recombination rates of the *R26-LSL-EYFP* allele; vessels were stained with anti-ERG (labels EC nuclei, for object segmentation), isolectinB4 (labels EC surface), and anti-GFP (detects YFP). Quantification of the contribution of EYFP^+^ ECs (ERG^+^, isolectinB4^+^) to arteries (indicated with a red A), arterial length, and vascular density reveals no significant differences, indicating poor Notch1 deletion (**C**). (**D–F**) Representative confocal micrographs of P6 retinal vessels from control mice (**D**) and Notch1 LOF mice (**E**) with high recombination rates of the *iSuRe-HadCre* allele. Quantification of the contribution of MbTomato + ECs to arteries, arterial length, and vascular density shows a very significant difference, confirming complete deletion of the *Notch1* gene in MbTomato + cells in animals containing the *iSuRe-HadCre* allele. (**G**) Comparative analysis of the contribution of *R26-LSL-EYFP* (YFP+) and *iSuRe-HadCre* (MbTomato^+^) ECs to retinal arteries (indicated with a red A), versus all capillaries, confirming that only cells expressing the recombined *iSuRe-HadCre* allele have full deletion of Notch1 and cannot form arteries. (**H**) scRNAseq analysis of ECs collected from livers and hearts by FACS indicate strong *Notch1* deletion in cells expressing the *iSuRe-Cre* or *iSuRe-HadCre* alleles. (**I**) Representative confocal micrograph showing retinal vessels (IsolectinB4^+^, ERG^+^) and quantification of the contribution of MbTomato^+^ ECs to the entire vascular network versus arteries, confirming very efficient deletion of *Rbpj* in the large majority of MbTomato^+^ cells. Data are presented as mean values ± s.d. For statistics, see Source Data File 1. Scale bars, 200 μm.

To confirm the utility and broad applicability of the *iSuRe-HadCre* allele for reliable single cell or whole tissue loss-of-function genetics, we analysed deletion efficiency for a total of 13 different floxed genes, with a genetic distance between LoxP sites ranging from 1.5 to 4.8 kb (Supplementary Figure S6A). Recombination efficiency correlates negatively with the genetic distance between LoxP sites, but is also strongly influenced by the gene locus and its DNA sequence (6–8), being highly variable. In contrast to reporter alleles, a full gene deletion requires the deletion of two floxed alleles. We initially used the standard *Rosa26-Lox-Stop-Lox-EYFP* reporter allele, containing 2.2kb between LoxP sites, as an internal comparative reference. The *Rosa26-iSuRe-HadCre* and *Rosa26-Lox-Stop-Lox-EYFP* alleles were independently combined with the *Notch1^floxed^* (3.5 kb between LoxP sites for each allele) and *Cdh5-CreERT2* alleles, a single high 4-OHT dose was injected at P1, and retinas were collected at P6. *Notch1* is an essential gene for the formation of arteries during vascular development (32), and decreased Dll4-Notch signaling induces higher retinal vessel density (44). Nevertheless, wildtype and *Notch1^floxed^* mutants carrying the *Rosa26-Lox-Stop-Lox-EYFP* allele had similar percentages of YFP + arterial cells, artery length, or vessel density (Figure 6A–C), which could suggest a lack of NOTCH1 function in these processes, or a lack of *Notch1* deletion in YFP + cells. Importantly, in animals carrying the *Rosa26-iSuRe-HadCre* allele, only ∼1.5% of arterial cells expressed the MbTomato reporter, despite a very high frequency of MbTomato^+^ cells in neighbouring capillaries and veins (Figure 6D–F). These data indicate that the efficiency of *Notch1^floxed^* deletion is very high only in animals and cells expressing the *Rosa26-iSuRe-HadCre* allele. This allowed us for the first time to uncover the full extent of the retinal *Notch1* loss-of-function phenotype. The observed increase in vascular density after deleting *Notch1* (Figure 6E) is significantly more pronounced than published before (45,46), suggesting incomplete *Notch1* deletion in all previous studies.

We also quantified the recombination efficiency in single cells of retinas exposed to a low dose of tamoxifen. Of the many ECs expressing the *iSuRe-HadCre* allele, all recombined the *Rosa26-Lox-Stop-Lox-EYFP* allele and none formed arteries, whereas many cells with recombination of only the *Rosa26-Lox-Stop-Lox-EYFP* reporter allele formed arteries, which were thus all false positives since they did not delete *Notch1* (Figure 6G). Single-cell RNAseq analysis of cells isolated by FACS confirmed the very efficient deletion of *Notch1*, and loss of its mRNA, in ECs collected 3 days after 4-OHT injection (Figure 6H). Overall, this data demonstrates that gene deletion with *iSuRe-HadCre* occurs quickly and is highly efficient, similar to *iSuRe-Cre*.

Next, we performed a similar analysis in mice carrying *Rbpj^floxed^* alleles. *Rbpj* is another gene essential for arterial development, and the very low percentage of MbTomato^+^ cells found in arteries confirmed that this gene was also very efficiently deleted in the large majority of cells recombining the *iSuRe-HadCre* allele (Figure 6I). Semi-quantitative PCR analysis in bulk FACS-sorted cells from postnatal and adult animals revealed that the *iSuRe-HadCre* allele enabled also highly efficient simultaneous deletion of *Notch1, Notch2*, and *Notch3* (6 floxed alleles) or of *Rbpj* and *Myc* in cells of different organs (Supplementary Figure S6B–H). Note that a small percentage of single cell events isolated by FACS will have contaminant wildtype cell RNA and DNA, as shown before (47), and these retain the non-recombined floxed genes.

**Figure 7.**
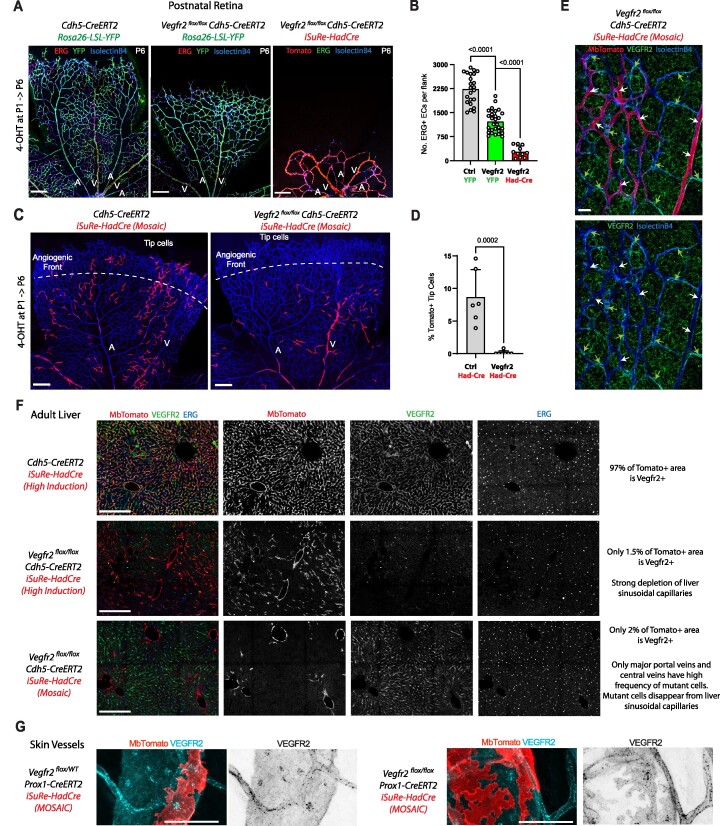
*iSuRe-HadCre* enables effective deletion of *Vegfr2* in the entire target tissue or in single cells. (**A**, **B**) Representative confocal micrographs of P6 retina vessels of control and *Vegfr2* floxed mice stained with anti-ERG (EC nuclei), IsolectinB4 (EC surface) and anti-GFP (detects YFP). Only animals containing the *iSuRe-HadCre* allele have complete and consistent deletion of the *Vegfr2* gene, resulting in a profound decrease in the number of blood vessels (including arteries (A) and veins (V)) and ERG + ECs. (**C**, **D**) Single cell mosaic analysis in retinas with low induction of the *iSuRe-HadCre* allele, showing that gene deletion is very effective in single cells and that single *Vegfr2* mutant cells cannot migrate to the angiogenic front (area above the dashed line) and do not form tip cells. (**E**) Immunostaining for VEGFR2 in iSuRe-HadCre mosaic retinas (note that VEGFR2 is expressed by ECs and many non-ECs in retinas) showing that MbTomato + cells (some indicated by white arrowheads) have deletion of *Vegfr2*, whereas many MbTomato negative cells (green arrowheads) do not have deletion of *Vegfr2*. (**F**) Confocal micrographs of liver cryosections showing the deletion of *Vegfr2/Kdr* in adult mice (8 weeks) liver ECs (Cdh5 + and ERG + nuclei). VEGFR2 immunostaining confirms the high deletion efficiency in MbTomato + cells of *Vegfr2* floxed mutants. Loss of VEGFR2 leads to the loss of liver sinusoids. Mosaic induction reveals that most *Vegfr2* mutant ECs survive in the larger portal veins and central veins vessels but not in sinusoidal capillaries. (**G**) Confocal micrographs of skin vessels of the indicated mice induced at P1 and P2 and collected at P21. Immunostaining with anti-VEGFR2 labels both lymphatic and blood endothelial cells. When recombination is mosaically induced by *Prox1-CreERT2*, only iSuRe-HadCre+/MbTomato + lymphatic ECs of *Vegfr2* floxed mutants loose VEGFR2 expression (see also Supplementary Figure S7). Scale Bars 200μm in A, C, F; 50 μm in E and G.

One of the most important genes for angiogenesis is *Kdr/Vegfr2*, that encodes for the most important VEGF receptor (VEGFR2). We have previously observed that is very difficult to delete this gene with the *Cdh5-CreERT2* allele during angiogenesis, presumably due to the floxed allele structure, but also due to the loss of the *Vegfr2* mutant ECs during angiogenesis, that are outcompeted by non-mutant cells over time (7,45). The *iSuRe-HadCre* allele allowed us to delete *Vegfr2* very effectively during retina angiogenesis, and in this way assess the real impact of fully deleting *Vegfr2* on vascular development (Figure 7A, B). Even when the induced recombination was very low, single *iSuRe-HadCre*/MbTomato + cells had deletion of VEGFR2, since they were excluded from the angiogenic front (Figure 7C, D). Immunostaining analysis confirmed the loss of VEGFR2 expression in *iSuRe-HadCre*/MbTomato+ ECs, but not in adjacent reporter negative cells (Figure 7E). Efficiency of deletion was also very high in *iSuRe-HadCre*/MbTomato+ adult liver ECs (Figure 7F). These are known to be very sensitive to the loss of VEGF/VEGFR2 signalling (48,49). When the *iSuRe-HadCre* allele was induced in mosaic fashion, most *Vegfr2* mutant (MbTomato+) ECs were depleted in the adult liver capillaries, and survived only in larger portal and central vessels (Figure 7F lower panel). In mosaic animals, the Cdh5-CreERT2+ ECs that did not undergo *iSuRe-HadCre* recombination, failed to delete *Vegfr2*, and formed most adult liver capillaries. Deletion of *Vegfr2* in lymphatic ECs with Prox1-CreERT2 was also very effective in iSuRe-HadCre/Tomato + cells, at both high and low (mosaic) recombination rates (Figure 7G and Supplementary Figure S7).

**Figure 8.**
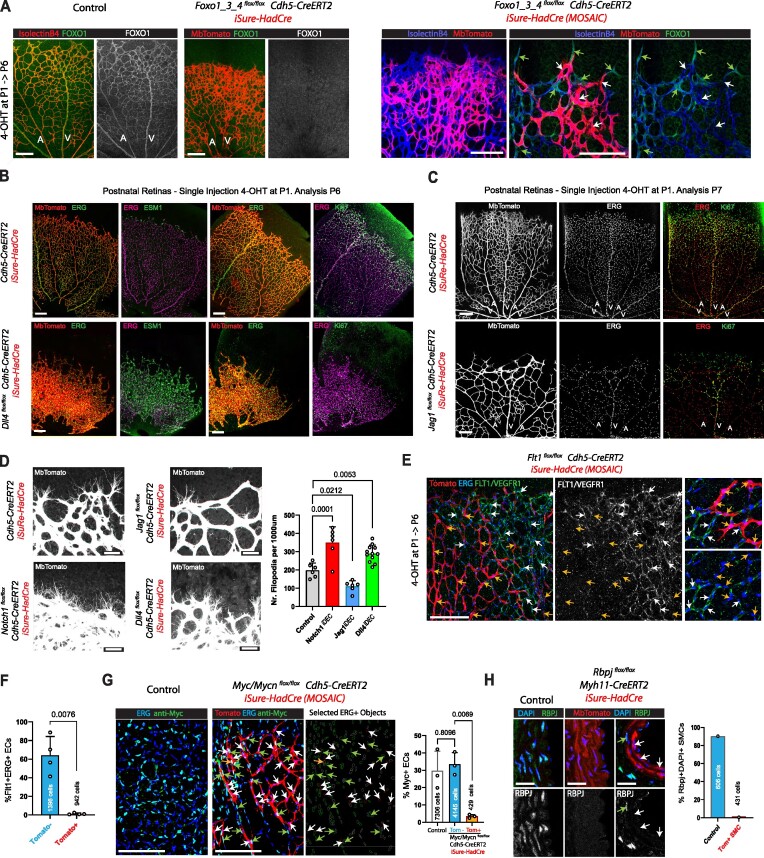
*iSuRe-HadCre* enables the deletion of multiple floxed genes in the entire target tissue or in single cells. (**A**) Retina confocal micrographs showing effective deletion of *Foxo* genes in MbTomato+ ECs (FOXO1 imunostaining and phenotypes characteristic of full Foxos depletion, such as defective EC sprouting and dense vasculature). (**B**) Confocal micrographs showing that one single injection of 4-OHT at P1 efficiently recombines *iSuRe-HadCre* in all retina ECs and deletes *Dll4*, leading to the strong upregulation of the tip cell marker gene Esm1 in most cells, and cell-cycle exit. ERG + ECs become Ki67- at the angiogenic front. (**C**) One single injection of 4-OHT at P1 efficiently recombines *iSuRe-HadCre* in all retina ECs and deletes the gene *Jagged1*, leading to the strong inhibition of angiogenesis. (**D**) The membrane-tagged Tomato protein from *iSuRe-HadCre* allows visualization of filopodia by confocal microscopy. Deletion of the indicated genes change the number of filopodia per vessel length. (**E**, **F**) Effective deletion of *Flt1*/*Vegfr1* is indicated by the loss of FLT1 protein in MbTomato+ ECs from the indicated mice. Note that Flt1 antibody signal is noisy. Specific signal is perinuclear/cytoplasmic. (**G**) Retina confocal micrographs of animals with mosaic induction of the *iSuRe-HadCre* allele showing that *Myc* and *Mycn* (both detected with Anti-Myc) are only effectively deleted in cells expressing the *iSuRe-HadCre* allele (MbTomato + cells, white and orange arrows). (**H**) Confocal micrographs of mouse aortas showing the effective deletion of *Rbpj* in MbTomato + of SMCs (Myh11+). White arrows indicate some nuclei/cells with MbTomato expression and lacking RBPJ, and green arrows without MbTomato expression and retaining RBPJ expression. Histogram showing the % of cells expressing RBPJ. Data are presented as mean values ± s.d. For statistics, see Source Data File 1. Scale Bars 200μm, except D, 50 μm and H, 25 μm.

To further demonstrate the efficiency of the *iSuRe-HadCre* allele, we determined the rate of genetic deletion and performed phenotypic analysis of postnatal and adult mice carrying multiple other floxed genes. Immunostaining and confocal analysis revealed a very effective deletion of all these genes in *iSuRe-HadCre* expressing cells, but not in cells lacking the expression of this allele (Figure 8). Simultaneously deletion of three *Foxo* genes, shown before to partially compensate for each other (23), is very efficient with *iSuRe-HadCre* after a single injection of 4-OHT (Figure 8A, left). The analysis of genetic mosaics showed that many of the Cdh5-CreERT2+ ECs failed to delete these 3 Foxo genes. On the other hand, iSuRe-HadCre/MbTomato+ ECs effectively deleted *Foxos* and did not form tip cells when surrounded by wildtype cells (Figure 8A, right). *Jagged1* and *Dll4* were also effectively deleted in animals containing the *iSuRe-HadCre* allele after a single dose of 4-OHT (Figure 8B, C), unlike the three doses of tamoxifen (P1, P2, P3) used before to delete these genes (50). Dll4 loss induced vascular hyperplasia, associated to cell-cycle arrest (Ki67-), increased filopodia (Figure 8D), and the upregulation of the tip cell marker Esm1, whereas Jagged1 loss strongly inhibited angiogenesis, as shown previously (44,50). *Flt1/Vegfr1* was also only effectively deleted in iSuRe-HadCre/MbTomato+ cells (Figure 8E, F). We found that for some genes, like *Myc* and *Mycn*, their protein is detectable in only around 29% of wildtype ECs during angiogenesis, being impossible to determine where are the mutant cells without the use of *iSuRe-HadCre* (Figure 8G). Simultaneous deletion of these two genes, that correspond to deletion of 4 alleles containing 4.6kb and 4.8kb of DNA between LoxP sites (Supplementary Figure S6A), was very effective only in iSuRe-HadCre/MbTomato + cells (Figure 8G). This data shows how the iSuRe-HadCre allele can be very relevant for multiple gene deletions in whole tissues or single cells, a method essential for epistasis analysis in mice.

We also tested the efficiency of *iSuRe-HadCre* with the *Myh11-CreERT2* line that is expressed in SMCs. iSuRe-HadCre + cells effectively deleted *Rbpj* in SMCs (Figure 8H).

Overall, these data confirm that when cells recombine and express the *iSuRe-HadCre* allele, even after a single and low tamoxifen pulse, they express enough Cre to efficiently recombine other floxed genes. In contrast, conditional genetics without using this allele is blind, even when using standard reporter alleles, because cells often do not recombine the intended floxed genes, thus generating many false positives that prevent the accurate determination of a gene function in single cells or entire tissues.

### Transient Cre expression driven by *iSuRe-HadCre* is non-toxic

**Figure 9.**
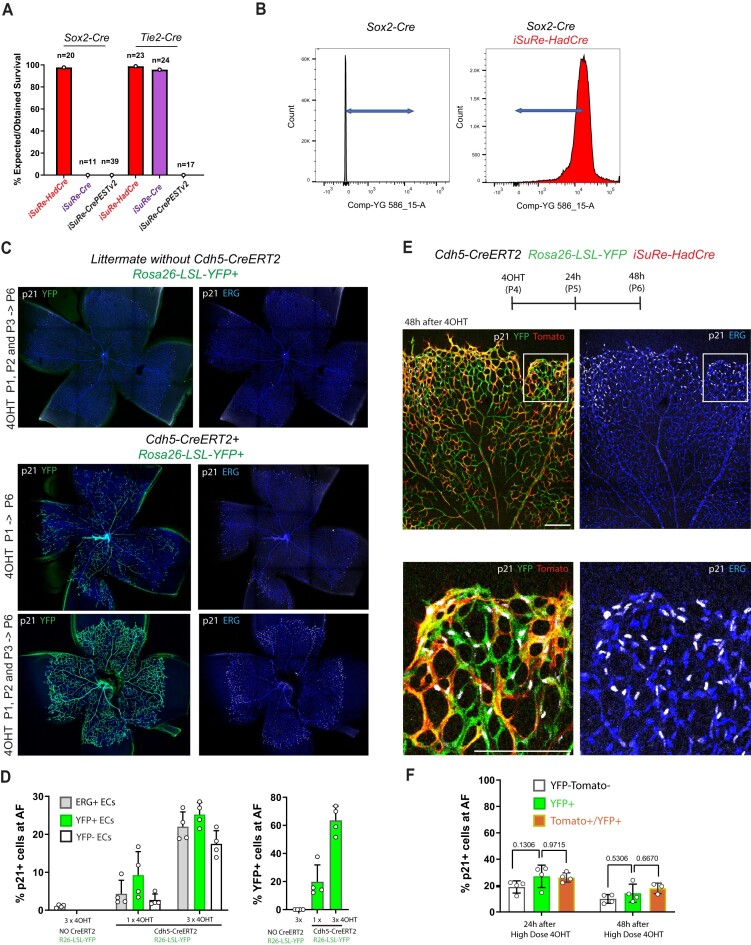
Expression of the *iSuRe-HadCre* allele does not elicit cellular toxicity. (**A**) Comparative analysis of survival rates of C57Bl6 animals containing the *Sox2-Cre* (recombines all cells), or *Tie2-Cre* (recombines only ECs and blood) alleles when combined with the different *iSuRe-Cre* lines. Only the *iSuRe-HadCre* is devoid of toxicity. (**B**) Histogram plot showing intensity of MbTomato signals in all liver cells of adult *Sox2-Cre iSuRe-HadCre* mice, confirming that the expression of the recombined allele is ubiquituous, permanent and non-toxic. (**C**, **D**) Confocal analysis of retinas from pups induced at P1, P2 and P3 (or only P1) and collected at P6. Cellular toxicity can be scored by the expression of the replicative stress or cell senescence marker p21 in ERG + ECs. The expression of this marker is much higher in retinas from animals containing the Cdh5-CreERT2 allele, and among these in retinas having a higher rate of recombination of the Rosa26-LSL-YFP allele (injected with 3 times more tamoxifen). (**E**, **F**) Short-term (24h and 48h after 4-OHT) analysis of the toxicity marker p21 in angiogenic front (AF) ECs expressing the *Rosa26-LSL-YFP* (YFP+) or the *iSuRe-HadCre* (MbTomato+/YFP+) allele reveal no additional cellular toxicity by the transient expression of Cre in the first 24–48 h after 4-OHT. Boxed areas showed at higher magnification below. Data are presented as mean values ± s.d. For statistics, see Source Data File 1. Scale bars, 200 μm.

We assessed toxicity induced by expression of the *Rosa26-iSuRe-HadCre, Rosa26-iSuRe-CrePEST^v2^* and *iSuRe-Cre* alleles in mice carrying these alleles on the C57Bl6 background. Only animals containing the *Rosa26-iSuRe-HadCre* allele survived upon recombination with both the *Tie2-Cre* allele (blood cells and ECs) and the *Sox2-Cre* allele (all embryo cells). These expressed the allele in all target cells (Figure 9A, B), confirming the lack of toxicity in cells with iSuRe-HadCre recombination and expression.

Given its higher sensitivity and recombination efficiency, the *Rosa26-iSuRe-HadCre* allele can also be used to significantly reduce the cellular toxicity associated with the consecutive tamoxifen injections usually required for conditional genetics analysis. In line with previous studies describing cell toxicity and impaired angiogenesis when high doses of tamoxifen are delivered to activate CreERT2 (51,52), we found that 4-OHT induces p21+ arrested/senescent ECs when animals express CreERT2 and display high rates of recombination of the *Rosa26-Lox-Stop-Lox-EYFP* reporter (Figure 9C, D). To more precisely determine the potential short-term cell toxicity arising just after *iSuRe-HadCre* allele activation, we compared the frequency of p21 expression in cells recombining the *Rosa26-Lox-Stop-Lox-EYFP* or *Rosa26-iSuRe-HadCre* alleles 24 and 48 h after tamoxifen injection. During this period, cells recombining the *iSuRe-HadCre* allele will have strong but transient Cre expression, whereas cells exclusively recombining the *Rosa26-Lox-Stop-Lox-EYFP* allele will only have tamoxifen-induced CreERT2. The additional transient Cre expression driven by the *Rosa26-iSuRe-HadCre* allele had no significant impact on p21 expression at 24 or 48 h (Figure 9E, F), despite the very efficient deletion of floxed alleles after a single injection of tamoxifen (Figures 6–8). This data suggest that the toxicity induced by tamoxifen in cells expressing CreERT2 is independent of canonical or productive Cre recombination. This is in line with previous reports showing that tamoxifen administration to animals expressing CreERT2 is more toxic than Cre expression itself (38,51). Given that the *Rosa26-iSuRe-HadCre* allele increases the efficiency of gene deletion without increasing cellular toxicity, it can be used to efficiently delete genes even at lower doses of tamoxifen, and in this way prevent toxicity associated to the use of high doses of tamoxifen to induce CreERT2-dependent floxed gene deletions.

## Discussion

Conditional Cre genetics has been used for decades and remain the gold standard for the analysis of gene function (1,3,39). Recent CRISPR/Cas9-based gene targeting approaches do not match it for genetic deletion efficiency and precision, especially in inducible genetic studies. However, despite the clear advantages of the Cre/Lox system, its temporal control and efficiency varies greatly with different Cre transgenes and floxed alleles (2,5,6,53), and this significantly decreases its reliability, particularly when applied to mosaic or single-cell genetics analyses that require lower or incomplete CreERT2 activity.

*iSuRe-Cre* was the first CreERT2-inducible, dual reporter-Cre-expressing mouse allele that overcame the problem of false positives in conditional Cre genetics (7). However, this first-generation transgene also had several caveats, including leakiness, relatively low sensitivity to Cre/CreERT2 activity, and the risk of toxicity in cells with high and permanent Cre expression (7). We found now that this toxicity varies across cell types, developmental stages, and mouse genetic backgrounds, as reviewed recently (38). All of these variables are difficult to control and predict when using different mouse models containing the *iSuRe-Cre* allele, and can impact the final phenotype of the analyzed cells.

To overcome these caveats, we set out to develop and characterize several new mouse lines, at each step encountering problems with the improved designs. The *iSuRe-CrePEST^v1^* line overcame the leakiness of *iSuRe-Cre*, particularly in myocytes, but suffered from the same low sensitivity to CreERT2 recombination and did not provide certainty that reporter-expressing cells had also recombined other floxed alleles. The *Rosa26-iSuRe-CrePEST^v2^* allele was non-leaky and significantly more sensitive to CreERT2 because of its location in the *Rosa26* locus, but the CAG-promoter expression from this locus was too high, and the very high and permanent co-expression of the reporter and CrePEST still induced significant toxicity in most tested cell types.

These unexpected results prompted us to design a similar Rosa26-targeting construct that would maintain non-leakiness and the high sensitivity to CreERT2 induction, but that at the same time would only support the induction of transient, but sufficient, expression of Cre. The solution we devised was to engineer a construct enabling the co-expression of Cre at equimolar levels with the much weaker recombinase FlpO(43), which would, after a delay, switch off the system and turn on the expression of a fluorescent reporter. In this way, the reporter would label cells that definitely had, but no longer have, high Cre (and FlpO) expression, as indicated by the name, *iSuRe-HadCre*. This new design and strategy proved to be effective and overcame all the problems we encountered with the previous models. In contrast with its predecessors, the *iSuRe-HadCre* allele is located in the ubiquitously expressed *Rosa26* locus, shows no leakiness, is highly sensitive to CreERT2 activity, supports higher reporter expression, has no Cre-related toxicity, and still ensures a very high rate of floxed gene deletion (true positives). Overall, we tested the efficiency of *iSure-HadCre* with *UBC-CreERT2, Cdh5-CreERT2, Prox1-CreERT2* and *Myh11-CreERT* that recombine most cell types, and with 13 different floxed genes. Deletion of all these genes was very effective with *iSuRe-HadCre*, even when multiple genes containing very large floxed cassettes (up to 4.8 kb) had to be deleted simultaneously. This is essential to perform reliable conditional functional genetics, particularly genetic epistasis analysis.

Is known that different floxed genes have very different sensitivities to Cre/CreERT2 activity, and their recombination rate may also vary with the cellular stage or differentiation state. Even the same gene may be differentially recombined depending on the cell type and the chromatin conformation of its locus. Genes not expressed in a given cell type and present in heterochromatin regions, may be also more difficult to delete by Cre, but they are in principle also less relevant to conditionally delete. In this work, we analysed multiple genes deletion rates across several cellular stages (embryonic, neonatal and adult) and in different cell types, and have not found significant differences in the efficiency of *iSuRe-HadCre*. Future work may address if higher or permanent Cre expression is necessary to fully delete some other floxed genes, at the cost of potentially causing cellular toxicity, as observed with the first generation *iSuRe-Cre*.

New tools that enhance the reliability of conditional genetics are gaining importance as technology moves towards single-cell mosaic genetics, which, when applied *in situ*, requires low tamoxifen doses or mosaic CreERT2 induction in relatively few cells. We have shown that the *iSuRe-HadCre* allele guarantees a very high rate of true positives in single induced cells. This allele allowed us to confirm how single cells losing *Notch1, Rbpj, Kdr* and *Foxo1/3/4* change their mobilization or differentiation behaviour across a growing vascular bed. Standard reporters and tools did not enable the induction and identification of cells with deletion of these genes, preventing determination of their important role in these processes.

Single-cell technologies are now standard for profiling control and mutant cells *ex situ*. Given that current single-cell sequencing technologies only detect the mRNA of some genes in single cells, and that most floxed genes conserve their non-floxed DNA and mRNA sequences after deletion, this often generates profiles of pseudomutant cells whose true genetic status cannot be determined. Of the 13 floxed genes analysed, 7 conserve their 3′mRNA after Cre-deletion and therefore their deletion cannot be determined by scRNAseq. Some antibodies we used also failed to detect the partial protein deletion by immunostaining given that they detect the protein upstream or downstream of the deletion. We have provided multiple evidence here that many of the CreERT2 expressing cells do not have deletion of the intended floxed genes after tamoxifen, indicating that the *iSuRe-HadCre* allele is necessary for the reliable isolation of *bona fide* mutant cells for reliable single-cell functional omics.

In addition to providing a new mouse model, we also derived embryonic stem cells from *iSuRe-HadCre* mice with or without the *UBC-CreERT2* and *Cdh5-CreERT2* alleles (Supplementary Figure S8). These can be easily modified by CRISPR/Cas9 to include other floxed alleles (54), and in this way perform reliable single cell conditional genetics in all differentiated cell lineages *in vitro*. The novel *iSuRe-HadCre* recombinase genetic cascade ensuring high transient Cre activity without toxicity can also be engineered in other cell lines or animal models. Given its ease of use, high efficiency, and lack of toxicity, we believe the *iSuRe-HadCre* allele will be essential for laboratories conducting conditional gene-function analysis with the Cre/lox system.

## Supplementary Material

gkae472_Supplemental_Files

## Data Availability

RNA-seq data can be viewed at the Gene Expression Omnibus (GEO) under accession number GSE245726. Instructions and code to reproduce all scRNA-seq results can be found at https://doi.org/10.5281/zenodo.11220492. All other data supporting the findings in this study are included in the main article and associated files.

## Notes

Present address: Anahita Hamidi, Uppsala University, Department of Immunology, Genetics and Pathology, Husargatan 3, 75122 Uppsala, Sweden.
