# Supplementary material for: iSuRe-HadCre is an essential tool for effective conditional genetics
Source: Nucleic Acids Res. 2024 Jun 8;52(13):e56. doi: 10.1093/nar/gkae472 (PMC11260470; doi:10.1093/nar/gkae472)
Supplement: gkae472_Supplemental_Files [file gkae472_supplemental_files.zip › Supplementary Data NAR FINAL file v2.pdf]

Supplementary Information

iSuRe-HadCre is an essential tool for effective conditional genetics

Irene Garcia-Gonzalez[1], Susana F. Rocha[1], Anahita Hamidi[1, ‡], Lourdes Garcia-Ortega[1], Alvaro Regano[1], Maria S. Sanchez-Muñoz[1], Mariya Lytvyn[1], Aroa Garcia-Cabero[1], Sergi Roig-Soucase[1], Hans *Schoofs*[7], *Marco Castro[2,3], Helena Sabata[4], Michael Potente[2,3], Mariona Graupera[4,5,6] Taija Makinen[7,8,9], Rui Benedito[1,*]*

[1]Molecular Genetics of Angiogenesis Group. Centro Nacional de Investigaciones Cardiovasculares (CNIC), Madrid, Spain.

[2]Angiogenesis & Metabolism Laboratory, Berlin Institute of Health at Charité – Universitätsmedizin Berlin, Berlin, Germany

[3]Angiogenesis & Metabolism Laboratory, Max Delbrück Center for Molecular Medicine in the Helmholtz Association, Berlin, Germany

[4]Endothelial Pathobiology and Microenviroment Group, Josep Carreras Leukaemia Research Institute (IJC), 08916 Badalona, Barcelona, Catalonia, Spain

[5]Centro de Investigación Biomédica en Red de Cáncer (CIBERONC), Instituto de Salud Carlos III, Av. de Monforte de Lemos, 5, 28029, Madrid, Spain

[6]ICREA, Institució Catalana de Recerca i Estudis Avançats, Pg. Lluís Companys 23, Barcelona, Spain

[7]Uppsala University, Department of Immunology, Genetics and Pathology, Dag Hammarskjölds väg 20, 751 85 Uppsala, Sweden.

[8]University of Helsinki, Haartmaninkatu 8, 00014 Helsinki, Finland.

[9]Wihuri Research Institute, Haartmaninkatu 8, 00290 Helsinki, Finland.

‡ Present Address: Uppsala University, Department of Immunology, Genetics and Pathology, Husargatan 3, 75122 Uppsala.

*Correspondence: Rui.benedito@cnic.es

Contents

Supplementary Figures S1-S8 (Pages 2-10)

Supplementary Tables 1-2 (Pages 11-14)

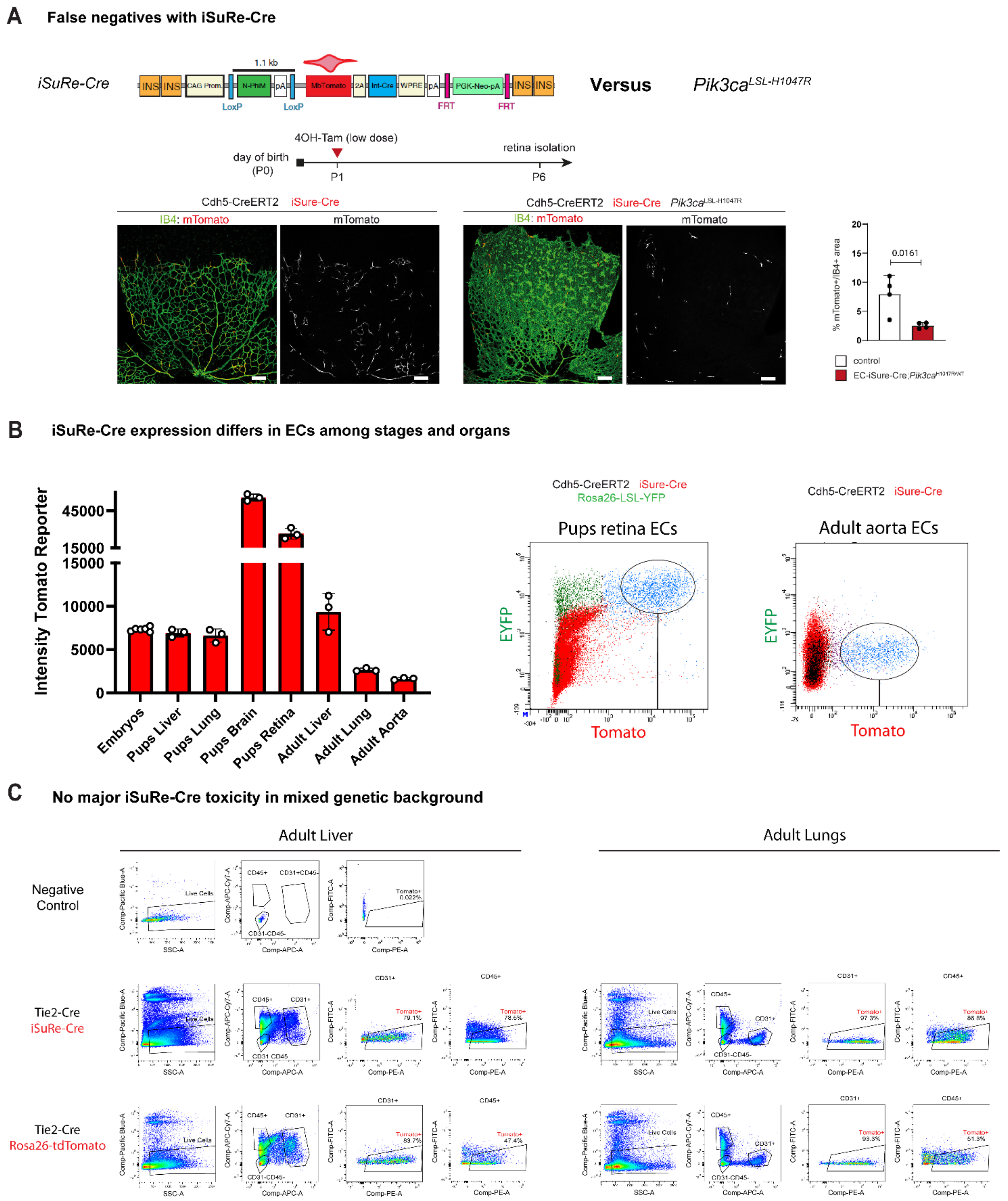

Supplementary Figure S1. Analysis of false negatives and expression or toxicity in *iSuRe-Cre* mice. (A) Mice containing the *iSuRe-Cre* genetic construct, the inducible floxed *Pi3KCA*^{H1047R} allele, and a *Cdh5-CreERT2* allele were induced with a low tamoxifen dose at P1, and retinas were collected for analysis at P6. Images show the relatively low recombination frequency of the *iSuRe-Cre* allele, with many false-negative cells having recombination of the *Pi3KCA*^{H1047R} allele and generating a strong vascular expansion/hyperdensity phenotype despite the scarcity of *iSuRe-Cre*$^{+}$ cells. **(B)** FACS analysis of the variability in *iSuRe-Cre/MbTomato-2A-Cre* expression levels in ECs collected at distinct developmental stages and from different organs. **(C)** Comparative FACS analysis of the recombination frequency and intensity of MbTomato-2A-Cre or TdTomato expression in ECs and blood lineages; MbTomato-2A-Cre expression is non-toxic when mice are on the C57Bl6 x CD1 genetic background. Data are presented as mean values ± s.d. For statistics, see Source Data File 1. Scale bars 150μm.

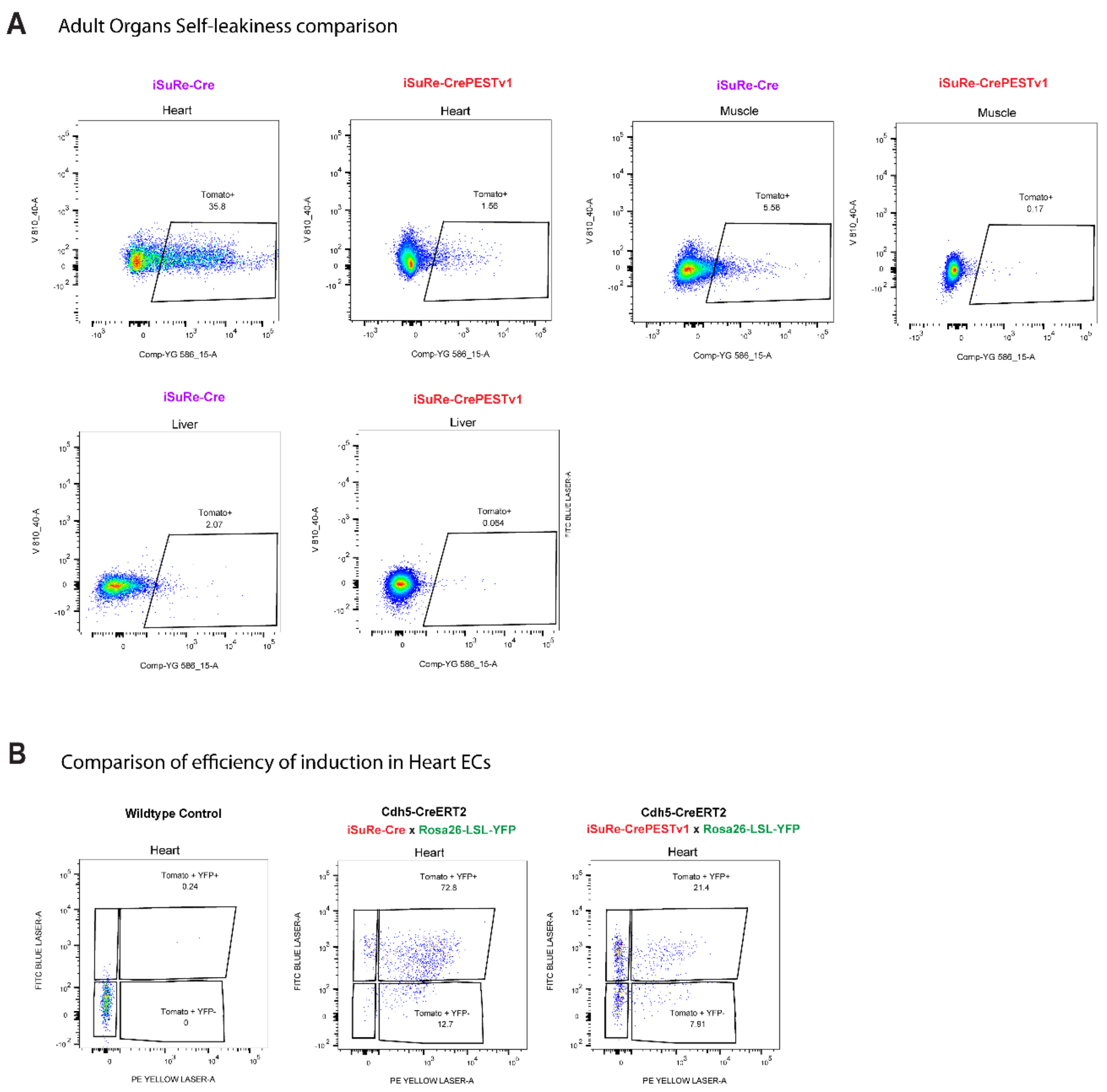

Supplementary Figure S2. The *iSuRe-CrePEST*^{v1} allele has lower self-leakiness and recombination efficiency than the *iSuRe-Cre* allele. (A) Comparative FACS analysis of *iSuRe-Cre* and *iSuRe-CrePESTv1* self-leakiness (indexed by the frequency of MbTomato^{+} cells) in different organs and in the absence of other CreERT2 alleles or induction. **(B)** Comparative FACS analysis of the recombination efficiency of the reference *Rosa26-LSL-YFP* allele in heart ECs (Cdh5-CreERT2^{+}) expressing the *iSuRe-Cre* or *iSuRe-CrePEST*^{v1} alleles. Efficiency is indexed as the percentage of MbTomato+YFP+ cells (true positives) to MbTomato+YFP- (false positives).

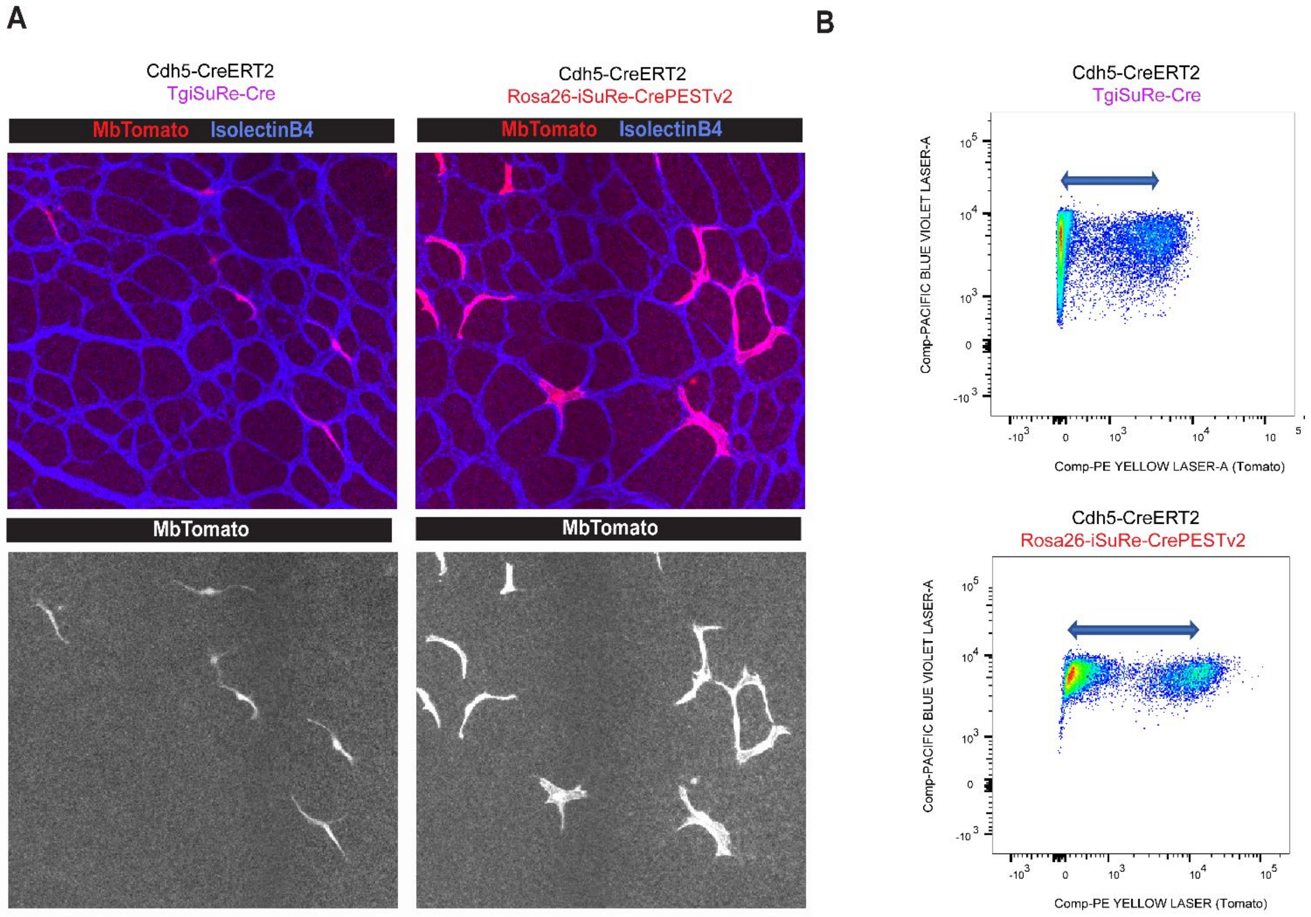

Supplementary Figure S3. The *Rosa26-iSuRe-CrePESTv2* allele is more highly expressed than previous *iSuRe-Cre* alleles. (A) Representative confocal micrographs of P6 retinas showing higher MbTomato fluorescence intensity in ECs (marked with IsolectinB4 and Cdh5-CreERT2$^+$) from animals expressing the *Rosa26-iSuRe-CrePESTv2* allele. **(B)** FACS plots showing higher MbTomato fluorescence intensity in postnatal day 7 liver ECs from animals expressing the *Rosa26-iSuRe-CrePESTv2* allele.

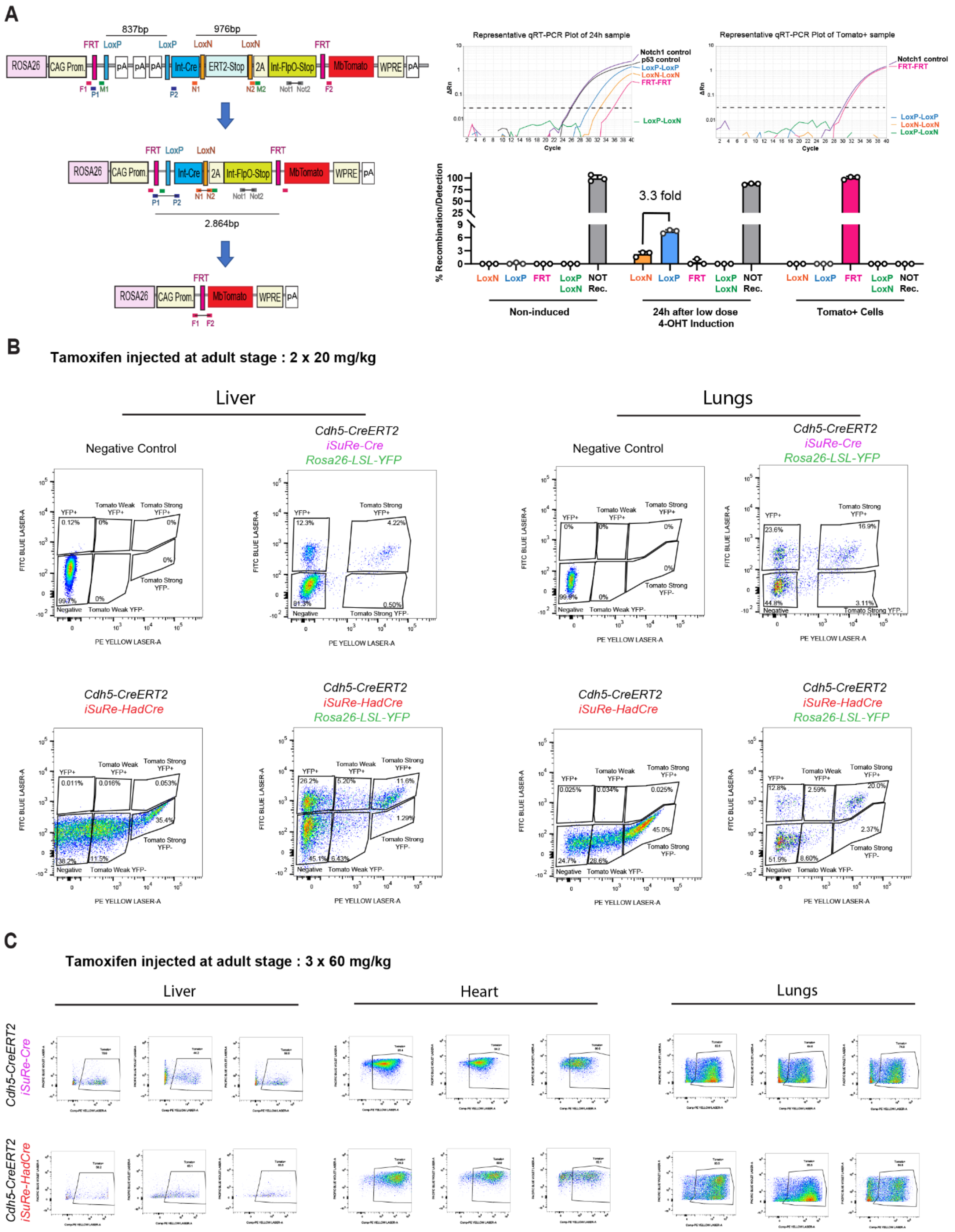

Supplementary Figure S4. Kinetics of the *Rosa26-iSuRe-HadCre* allele recombination and its efficiency in adult quiescent cells. (A) Predicted genetic recombination cascade and quantitative real time PCR with the indicated primers show how recombination between the LoxP sites is more effective than recombination

between the LoxN sites. It also shows that LoxP does not recombine with LoxN (green) and that once cells become Tomato+, the allele is fully recombined, and only a PCR with primers flanking the FRT site is amplified. **(B)** Comparative FACS analysis of recombination efficiency in Cdh5-CreERT2–induced liver and lung ECs reveals generally higher sensitivity of the *Rosa26-iSuRe-HadCre* allele to CreERT2 than *iSuRe-Cre*. MbTomato^{+} cells collected from iSuRe-Cre^{+} or iSuRe-HadCre^{+} animals reliably report recombination of the reference *Rosa26-LSL-YFP* reporter allele. (**C)** Comparative FACS analysis of recombination efficiency in adult quiescent ECs after three very high tamoxifen doses, showing similar recombination rates.

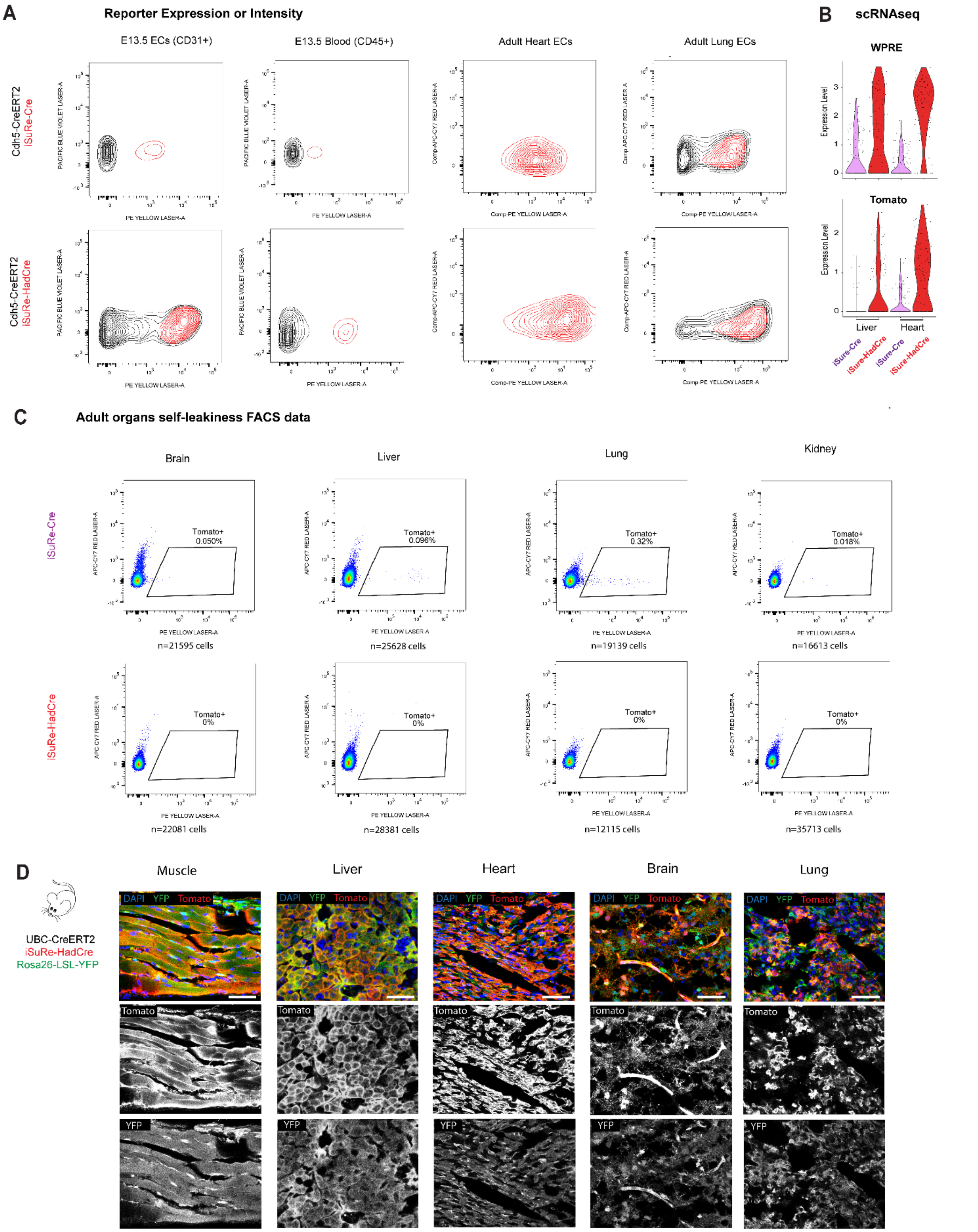

Supplementary Figure S5. The *Rosa26-iSuRe-HadCre* allele provides higher reporter expression and zero leakiness. (A) Comparative FACS analysis in different cell types and stages of development showing the higher expression of the reporter MbTomato (yellow laser axis) in *Rosa26-iSuRe-HadCre* animals. **(B)** scRNAseq analysis reveals higher expression of the *iSuRe-HadCre* allele in single cells. The WPRE and MbTomato sequences are the same in the two alleles. **(C)** Comparative FACS analysis showing that *Rosa26-iSuRe-HadCre* organs have 0% MbTomato+ cells (zero leakiness). **(D)** Histology analysis of the indicated animals showing very efficient recombination of the YFP reporter in all iSuRe-HadCre+/MbTomato+ cells. Scale Bars 50µm.

A

Floxed gene	*Notch1*	*Notch2*	*Notch3*	*Rbpj*	*Myc*	*Mycn*	*Foxo1*	*Foxo3*	*Foxo4*	*Kdr*	*Flt1*	*Dll4*	*Jag1*
Inter-LoxP distance (kb)	3.5	1.1	2.1	3	4.8	4.6	3.2	2.7	2.2	2.4	2.0	1.5	4.4

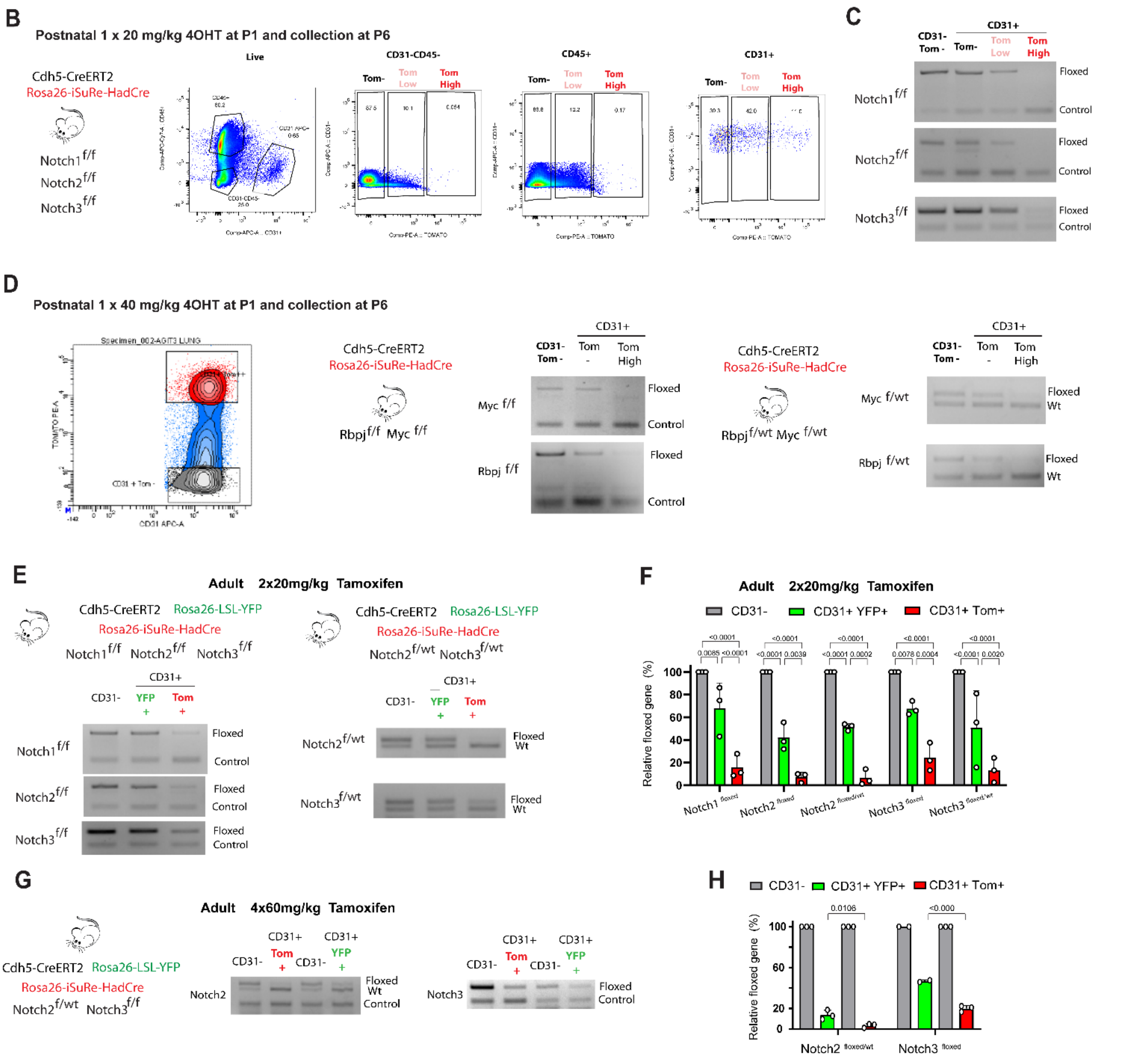

Supplementary Figure S6. The *Rosa26-iSuRe-HadCre* allele enables efficient deletion of multiple floxed alleles in postnatal and adult cells. (A) Distances between LoxP sites for all the floxed genes used in this study. **(B)** FACS plots with gating strategy used to isolate cells from the indicated animals. Control cells were CD31^{-}, Tom- (non-endothelial, Cdh5-CreERT2^{-}), and CD31^{+} cells were gated based on zero, low or high MbTomato content. Only MbTomato^{high} cells were specific to the endothelial lineage (CD31^{+}, Cdh5^{+}) and had recombination of the *iSuRe-HadCre* allele. **(C)** Semi-quantitative DNA PCR with primers detecting the floxed sequence and a reference wildtype sequence as control. The gels show a pronounced deletion of *Notch1, Notch2*, and *Notch3* floxed genes only in cells that had recombination of the iSuRe-HadCre allele (CD31+ MbTomato^{high}). **(D)** FACS plots with gating strategy used to isolate cells from animals with the indicated genotype. The lower PCR band served as an internal control. The PCR gels show pronounced deletion of *Rbpj* and *Myc* floxed genes only in CD31+ MbTomato^{high} cells. **(E-H)** Adult mice were induced for 2 or 4 consecutive days. CD31^{-} (Cdh5^{-}, negative control, non-induced), CD31^{+}YFP^{+}, and CD31^{+}MbTomato^{+} cells were collected and DNA extracted. Gel pictures show representative semi-quantitative PCR results, and charts show relative quantification. Data are presented as mean values ± s.d. For statistics, see Source Data File 1.

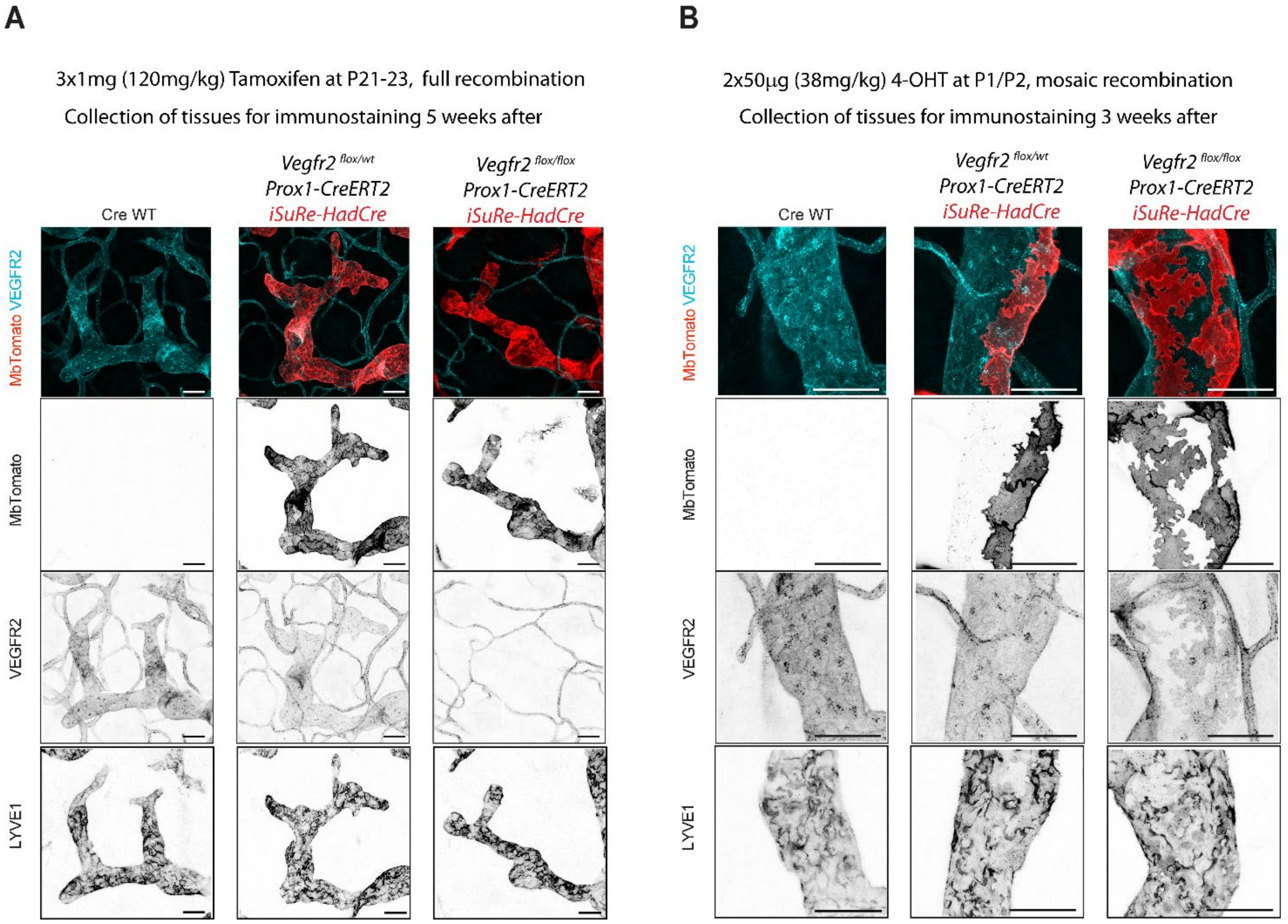

Supplementary Figure S7. Effective gene deletion in lymphatic endothelial cells with iSuRe-HadCre. (A, B) Representative confocal micrographs of skin vessels of control and *Vegfr2* floxed mice induced as indicated and stained with anti-VEGFR2 that labels both lymphatic (PROX1+LYVE1+) and blood endothelial cells (PROX1- LYVE1-). Anti-LYVE1 stains lymphatic endothelial cells. At high recombination rates (A), all lymphatic endothelial cells recombine the *iSuRe-HadCre* allele and delete VEGFR2. At low recombination rates (B), a mosaic of cells expressing *iSuRe-HadCre* is induced. MbTomato+ cells lose VEGFR2 expression, whereas the neighbouring cells retain the gene expression despite expressing *Prox1-CreERT2* and being induced with the same dose of tamoxifen. Scale Bars 50µm.

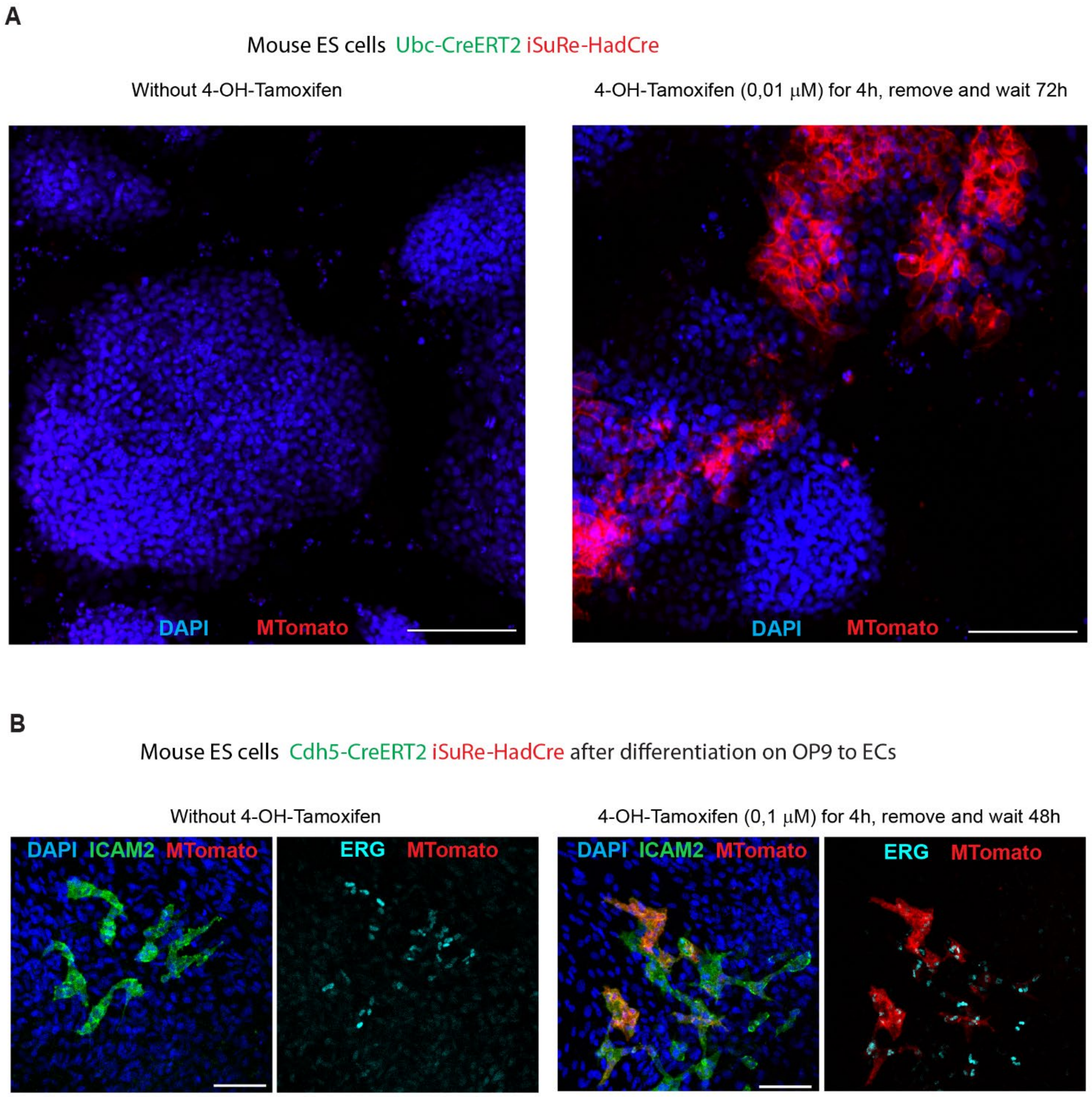

Supplementary Figure S8. Mouse ES cells from iSuRe-HadCre mice as an *in vitro* model for effective conditional genetics. (**A**) Representative confocal micrographs of the indicated ES cells showing the nuclei (DAPI+) and the membrane Tomato protein in induced cells (iSure-HadCre+), 72h after induction with a low dose of 4-OHT. (**B**) Representative confocal micrographs of ES cells with the indicated genotype co-cultured with OP9 cells for 8 days. After six days of coculture, ES cells differentiate into ECs: ICAM2+ (EC surface) and ERG+ (EC nuclei) and the addition of a relatively low dose of 4-OHT for 4h at day 6 of differentiation, was able to induce recombination of the *iSuRe-HadCre* allele and expression of the MbTomato reporter specifically in Cdh5+, ICAM2+, ERG+ endothelial cells. Scale bars 100 microns.

Supplementary Table 1. Genotyping primers

Name of mouse line	Expected size fragment	Genotyping oligonucleotides
Tg(PAC-Cdh5-CreERT2)^{1Rha}	*Allele band: 550bp*	Cdh5 Trans F: GGAGGCTGGAAAGTAGAGCA<br>CreM R: TCCCTGAACATGTCCATCAG
Tg(BAC-Myh11-CreERT2)^{1Soff}	*Allele band: 287bp*<br>*WT band: 200bp*	Rev Cre: AGT CCC TCA CAT CCT CAG GTT<br>Rev Myh11: AAC TCC ACG ACC ACC TCA TC<br>Myh11 Fwd: TGA CCC CAT CTC TTC ACT
Tg(Sox2-Cre)	*Allele band: 165bp*<br>*WT band: 207bp*	SOX2 WT Fwd: CTT GTG TAG AGT GAT GGC TTG A<br>SOX2 Common: TAG TGC CCC ATT TTT GAA GG<br>SOX2 Transgene Fwd: CCA GTG CAG TGA AGC AAA TC
Tg(iSuRe-Cre)	*Allele band: 260bp*<br>*WT band: 104 bp*	Sv40 pA F: CCCCCTGAACCTGAAACATA<br>AVT MTomato R: CCTTGCTCACCATGGTCTTG<br>Chr17 R: GTGTCTGTACCAGGTTGGTTTG<br>Chr17 R: GCTCTGCATGTTGCAAGAAA
Tg(Tie2-cre)	*Allele band: 500bp*	Ts: GGG AAG TCG CAA AGT TGT GAG TT<br>C2: CTA GAG CCT GTT TTG CAC GTT C
iSuRe-CrePESTv1	*Allele band: 1kb*	H2B F: CGAGGGTACTAAGGCCATCA<br>CUB end R: GGAATTCGGGGATCCCTAT
Rosa26-iSuRe-CrePESTv2	*WT band: 250bp*<br>*Allele band: 373bp*	Rr711:GCACTTGCTCTCCCAAAGTC<br>Rr713:CTTTAAGCCTGCCCAGAAGA<br>ERT2 Fwd: TCCTCATCCTCTCCCACATC<br>PEST Rev: atcatcctgctcctccacct
Rosa26-iSuRe-HadCre	*WT band: 250bp*<br>*Allele band: 354bp*	Rr713:CTTTAAGCCTGCCCAGAAGA<br>Rr711:GCACTTGCTCTCCCAAAGTC<br>iCreERT2 F: ATCGCATTCCTTGCAAAAGT<br>FlpO trans and seq Rev II: catccagcacaggtaggtca
R26-LSL-EYFP	*WT band: 250bp*<br>*Allele band: 350bp*	Rr711:GCACTTGCTCTCCCAAAGTC<br>Rr712: GGGCGTACTTGGCATATGAT<br>Rr713:CTTTAAGCCTGCCCAGAAGA<br>Rr714:GCGAAGAGTTTGTCCTCAACC
Gt(ROSA)26Sor$^{tm14(CAG-tdTomato)Hze}$	*WT band: 250bp*<br>*Allele band: 350bp*	Rr711:GCACTTGCTCTCCCAAAGTC<br>Rr713:CTTTAAGCCTGCCCAGAAGA<br>CAG PCR F: CGGGGTCATTAGTTCATAGCC<br>CAG PCR R: CACCTCGACCATGGTAATAGC
Gt(ROSA)26Sor$^{tm2(Pik3ca*)Dsa}$	*WT band: 330bp*<br>*Allele band: 630bp*	aIMR4349: CTCTGCTGCCTCCTGGCTTCT<br>aIMR4350: CGAGGCGGATCACAAGCAATA<br>XpGL3:TGAATAGTTAATTGGAGCGGCCGCAATA

		PI3K -Rev: AAATAGCCGCAGGTCACAAAGTCTCCG
***Pik3ca**$^{tm1.1Waph}$*	*WT band:553bp*<br>*Allele band: 601bp*	19F: TTGGTTCCAGCCTGAATAAAGC<br>20R: GTCCAAGGCTAGAGTCTTTCGG
Notch1** $^{flox/flox}$ **mice	*WT band: 445bp*<br>*Flox band: 500bp*	Notch1 lox F: CTGAGGCCTAGAGCCTTGAA<br>Notch1 lox R: TGTGGGACCCAGAAGTTAGG
Notch2** $^{flox/flox}$ **mice	*WT band: 202 bp*<br>*Flox band: 240 bp*	Notch2 rv: GAG CCT TTT CCC CAT ATT CC<br>Notch2 Fwd: CAA CCC CAG ATA GGA AGC AG
Notch3** $^{flox/flox}$ **mice	*WT band: 150bp*<br>*Flox band: 190bps*	Notch3 LoxP1 F: tggctatgaggaccaaaagg<br>Notch3 LoxP1 R: ctgcctctagcactgggttt
Rbpj** $^{flox/flox}$ **mice	*WT band: 200bp*<br>*Flox band: 350bp*	Rbpj lox F: ATAATTTGCCAAGCCAAAGC<br>Rbpj lox R: GCTCCCCACTGTTGTGAACT
Myc** $^{flox/flox}$ **mice	*WT band:315bp*<br>*Flox band: 370bp*	Myc Flox RB F: gacagggatgtgaccgattc<br>Myc Flox RB R: gctcagtctccggctatcac
Mycn** $^{flox/flox}$ **mice	*WT band: 200bp*<br>*Flox band: 220bp*	N-Myc 1: GTCGCGCTAGAAGAGCTGAGAT<br>N-Myc3: CACAGCTCTGGAAGGTGGGAGAAAGTTGAAGCGTCTCC
Dll4** $^{flox/flox}$ **mice	*WT band: 400bp*<br>*Flox band: 500bp*	Dll4 lox F: GTGCTGGGACTGTAGCCACT<br>Dll4 lox R: TGTTAGGGATGTCGCTCTCC
Jag1** $^{flox/flox}$ **mice	*WT band: 401bp*<br>*Flox band: 453bp*	Jag4716: AGG TTG GCC ACC TCT AAA TC<br>Jag5032: GCA AGT CTG TCT GCT TTC ATC
Foxo1** $^{flox/flox}$ **mice	*WT band: 115bp*<br>*Flox band: 149bp*	Foxo1 primer A: GCTTAGAGCAGAGATGTTCTCACATT<br>Foxo1 primer B: CCAGAGTCTTTGTATCAGGCAAATAA<br>Foxo1 primer D: CAAGTCCATTAATTCAGCACATTGA
Foxo3** $^{flox/flox}$ **mice	*WT band: 100bp*<br>*Flox band: 138bp*	Foxo3 primer A: ATTCCTTTGGAAATCAACAAAACT<br>Foxo3 primer B: TGCTTTGATACTATTCCACAAACCC<br>Foxo3 primer D: AGATTTATGTTCCCACTTGCTTCCT
Foxo4** $^{flox/flox}$ **mice	*WT band: 343bp*<br>*Flox band: 555bp*	Foxo4 primer A: CTTCTCTGTGGGAATAAATGTTTGG<br>Foxo4 primer B: CTACTTCAAGGACAAGGGTGACAG<br>Foxo4 primer D: TGAGAAGCCATTGAAGATCAGA
Kdr** $^{flox/flox}$ **mice	*WT band: 200bp*<br>*Flox band: 220bp*	Flk lox F3: GCCTGCAGGTTCTGGTTTG<br>Flk lox Rev1: GCTGTGACATCTGGGTAGAG
Flt1** $^{flox/flox}$ **mice	*WT band: 120bp*<br>*Flox band: 180bp*	VEGFR1 lox F: CCT GCA TGA TTC CTG ATT GGA<br>VEGFR1 lox R: GCC TAA GCT CAC CTG CGG
**Control for semi-quant. PCRs**	*WT band: 104bp*	R: GTGTCTGTACCAGGTTGGTTTG<br>F: GCTCTGCATGTTGCAAGAAA

Supplementary Table 2. Quantitative real-time PCR primers

(used in Sup. Fig. S4A data)

LoxN Flank N1: CCACTGCCGCTAGCTAATAA
LoxN Flank Probe: TGCTGGAAGATGGCGATCCGATAA
LoxN Flank N2: CCGTAACCTGGATAGTGAAACA
LoxP Flank P1: GGCCGAATTCGATCTAGCTT
LoxP Flank Probe: TTATTAGGTCCCTCGACCTGCAGC
LoxP Flank P2: GGCCCTAAGAAGTTCCTATTCTC
FRT Flank F1: TCACCATGGTCTTGGAGAAAC
FRT Flank Probe: TTGATTTGATACCGCGGGCCCTAA
FRT Flank F2: TGCTGGTTATTGTGCTGTCT
LoxP-LoxN Flank M1: CGACGTCACCGCATGTTA
LoxP-LoxN Flank Probe: AGTGGAGAGGGCAGAGGAAGTCT
LoxP-LoxN Flank M2: GGGCCCTAAGAAGTTCCTATTC
FlpO Not1: CTGTACCAGTTCCTGTTCCTG
FlpO Probe: TCTTGATGTCGCTGAACCTGCCG
FlpO Not2: CTTGTCTTGGTCTCGGTCAC
Notch1 genomic P1: CGTCCAAGATGCCTTCCTTAT
Notch1 genomic Probe: TGGAATCTGGAACGAACAACACCTCC
Notch1 genomic P2: GGGCCCTTATTCCTTGTTGA
P53 genomic P1: CTGTGGATGGGACAAAGAAGA
P53 genomic Probe: AGAGCAGAAAGGGACTTGGGCTTT
P53 genomic P2: TCCAGACTTCCTCCAGAAGATA

Supplementary Table 3. Antibodies

ANTIBODIES	APPLICATION	DILUTION	SOURCE	IDENTIFIER
Goat anti-GFP	IF	1:200	Acris Antibodies	Cat# R1091P
Chicken anti-GFP	IF	1:200	Abcam	Cat# ab13970
Rabbit anti-DsRed	IF	1:400	Clontech	Cat# 632496
Rabbit anti-RFP-CF594	IF	1:400	Biotium	Cat#20422
Mouse anti-V5-FITC	IF	1:100	ThermoFisher	Cat# R963-25
Mouse anti-V5-Alexa488	IF	1:100	Biorad	Cat#MCA1360A488
Rabbit Anti-V5	IF	1:200	Abcam	Cat# ab9116
Rabbit anti-ERG-AF-647	IF	1:200	Abcam	Cat# ab196149
Rabbit anti-ERG (EPR3863)	IF	1:200	Abcam	Cat# ab110639
Rat anti-Ki67e660	IF	1:200	eBioscience/Thermo	Cat# 50-5698-82
Rat anti-Endomucin	IF	1:200	Santa Cruz Biotechnology	Cat# SC-53941
Rat anti-Endomucin	IF	1:200	Santa Cruz Biotechnology	Cat# SC-65495
Rat anti-CD31	IF	1:200	BD Biosciences	Cat# 553370
Rat anti-p21 (monoclonal)	IF	1:10	CNIO	
Rat anti-VEGFR2/KDR	IF	1:50	BD Pharmingen	Cat# 666307
Rabbit anti-Myc	IF	1:200	Millipore	Cat# 06-340
Rat anti-RBPJ	IF	1:50	Cosmobio	Cat# SIM-2ZRBP2
Goat anti-Esm1	IF	1:200	R&D systems	Cat# AF1999
Rabbit anti-FOXO1	IF	1:200	CellSignalling Technology	Cat# 2880S
Rat anti-FLT1	IF	1:200	ImClone	Cat# MF-1
Rat anti-ICAM2	IF	1:200	BD Biosciences	Cat# 553325
Rat anti-CD31-APC	FACS	1:200	BD Pharmingen	Cat# 551262
Rat anti-CD45-APC-Cy7	FACS	1:200	BD Pharmingen	Cat# 557659
Donkey anti-rat Alexa-647	IF	1:400	Abcam	Cat# ab150155
Donkey anti-goat Alexa-488	IF	1:400	Invitrogen	Cat# A-11055
Donkey anti-rabbit Alexa-594	IF	1:400	Jackson Immunoresearch	Cat# 711-587-003
Donkey anti-rabbit Alexa-647	IF	1:400	Jackson Immunoresearch	Cat# 711-607-003
Biotin-SP Donkey anti-goat	IF	1:200	Jackson Immunoresearch	Cat# 705-065-003
Biotin-SP Donkey anti-rat	IF	1:100	Jackson Immunoresearch	Cat# 712-067-003
